# Impact of Gut Microbiota on the Association between Diet and Depressive Symptoms in Breast Cancer

**DOI:** 10.3390/nu14061186

**Published:** 2022-03-11

**Authors:** Gusonghan Maitiniyazi, Xiaoyun Cao, Yue Chen, Rong Zhang, Yuan Liu, Ziyuan Li, Danfeng Gu, Tong Li, Shufang Xia

**Affiliations:** 1 Wuxi School of Medicine, Jiangnan University, Wuxi 214122, China; gusonghanmaitiniyazi@stu.jiangnan.edu.cn (G.M.); chenyue@stu.jiangnan.edu.cn (Y.C.); liuyuan@stu.jiangnan.edu.cn (Y.L.); liziyuan@stu.jiangnan.edu.cn (Z.L.); 2 Department of Breast Surgery, The Affiliated Wuxi Matemity and Child Health Care Hospital of Nanjing Medical University, Wuxi 214002, China; caoxiaoyun@njmu.edu.cn; 3 Department of Breast Surgery, Affiliated Hospital of Jiangnan University, Wuxi 214125, China; zhangrong@jiangnan.edu.cn (R.Z.); gudanfeng@jiangnan.edu.cn (D.G.); 4 Department of Psychiatry, Jiangsu Rongjun Hospital, Wuxi 214035, China; litong2202@163.com

**Keywords:** breast cancer, depression, dietary intake, gut microbiota, diet quality, nutrients

## Abstract

Little is known about the relationship between diet and depression through the gut microbiota among breast cancer patients. This study aimed to examine the dietary intake differences between depressed breast cancer (DBC) and non-depressed breast cancer (NBC) patients, and whether the differences could lead to gut microbiota changes that affect depressive symptoms. Participants completed the Center for Epidemiological Studies-Depression Scale (CES-D) and 24 h dietary recall. Fecal samples of 18 DBC patients and 37 NBC patients were collected for next-generation sequencing. A total of 60 out of 205 breast cancer patients reported significant depressive symptoms suggested by a CES-D score ≥ 16, which might be related to lower intakes of energy, protein, dietary fiber, vitamin A, vitamin B2, niacin, calcium, phosphorus, potassium, iron, zinc, selenium, manganese and tryptophan, and a poor diet quality indicated by a lower total Chinese Healthy Eating Index (CHEI) score. Additionally, NBC patients demonstrated greater gut microbiota diversity and a healthier composition, in which the relative abundances of Proteobacteria and *Escherichia-Shigella* were both lower than in the DBC patients (*p* < 0.05). Alpha diversity was a significant mediator between diet quality and depression, while calcium, phosphorus and selenium significantly regulated depression independent of the gut microbiota. Breast cancer-related depressive symptoms might be associated with a poor diet quality via gut microbiota-dependent pathways and lower micronutrient intake via microbiota-independent pathways.

## 1. Introduction

Breast cancer has become the most commonly diagnosed cancer among women worldwide, with approximately 2.26 million newly diagnosed cases and 685,000 deaths in 2020 [1]. Studies have shown that the disease diagnosis and treatment might cause psychiatric comorbidities in breast cancer patients, such as emotional and cognitive disorders [2]. As one of the most common psychiatric symptoms, the incidence of depression in breast cancer survivors (ranging from 9.4% to 66.1%) was much higher than in women without cancer [3,4]. Depression can lead to severe physical symptoms, raised functional impairment and poor treatment adherence and is an independent predictor of breast cancer recurrence and survival [5].

Accumulated evidence suggested that the factors leading to depression in breast cancer patients included demographic and social, disease, psychological and behavioral factors [2,6], in which the avoidance of lifestyle risk factors (especially diet) might lead to a reduction in depression [7]. Diet has been recognized as a critical regulator of the occurrence and progression of mental illness [8]. Better diet quality or healthy dietary patterns were related to lower depressive symptoms [9,10]. A balanced dietary pattern (e.g., the Mediterranean diet) and some foods (e.g., fish, fresh vegetables and fruits) have been related to a lower risk of depression or depressive symptoms; conversely, a high-fat Western diet and sugar-sweetened beverages have been considered as the risk factors [11]. However, the reality is that breast cancer patients rarely get professional dietary advice after their diagnosis, and some patients may have nutritional problems such as insufficient nutrient intake and an unreasonable dietary structure, which may increase the risk of depression. Tryptophan (TRP), a precursor of serotonin (also known as 5-hydroxytryptamine, 5-HT), must be obtained from the diet and is implicated in the serotonin-dependent pathophysiology and treatment of depression [12]. Due to the characteristics of malignant tumors and the use of some chemotherapy drugs, cancer patients demonstrated significantly lower 5-HT levels [13], and the 5-HT levels showed a negative association with self-reported levels of depression [14]. Changing the dietary structure or diet quality can affect the ratio of tryptophan to large neutral amino acids (LNAAs) to control the content of tryptophan entering the brain, and subsequent 5-HT synthesis to regulate depression [15]. 

As an essential modulator of gut–brain axis interaction, the gut microbiota, a consortium of microbes inhabiting the gastrointestinal tract, can affect the synthesis of 5-HT in the brain via immunological, neuroendocrine and direct neural mechanisms, as well as controlling tryptophan metabolism in the gut [16]. Consequently, the microbial influence on tryptophan metabolism and the serotonergic system might be critical in regulating brain function and behaviors [17]. External factors such as diet, antibiotics and probiotics affect the gut microbiota composition and metabolism, in which diet has been recognized as one of the leading environmental factors driving the composition and metabolism of the colonic microbiota [18]. Compared with short dietary interventions, long-term dietary habits with a specific composition of nutrients, meal times and eating behaviors lead to deeper and chronic changes in the gut microbiota [19]. Western diet-induced gut microbiota dysbiosis has been verified to damage human digestive physiology, have pathogenic influences on the immune system and subsequently increase neuroinflammation [20]. Given that the gut microbiota evolves with the digestive, immune and nervous systems of the host, diet alterations will probably have a prominent impact on the gut microbiota and depression.

A variety of studies focused on depression, dietary intake and the gut microbiota have been conducted, and strong evidence indicates a close correlation between diet quality and depression. However, for breast cancer patients, studies that explore the relationship between diet and depression through the gut microbiota are lacking. Accordingly, the objective of this study was to investigate the relationships among diet, the gut microbiota and depressive symptoms in breast cancer patients by conducting a cross-sectional study of dietary intake, fecal microbial taxa abundances and depressive status, to provide dietary evidence for the prevention and treatment of depressive symptoms in breast cancer patients.

## 2. Materials and Methods

### 2.1. Participants and Study Design

This study was a cross-sectional study (Chinese Clinical Trials Registry ChiCTR2100043177) that recruited 205 subjects diagnosed with breast cancer (30–79 years of age) at Wuxi Maternal and Child Health Care Hospital and Jiangnan University Affiliated Hospital from September 2020 to September 2021. The study was approved by the Medical Ethics Committee of Jiangnan University (JNU20200927RB01). Participants were screened based on the following inclusion criteria: female breast cancer without metastasis and diffusion; aged between 18 and 80 years old; Karnofsky performance status (KPS) ≥ 70; normal cognitive function and reading ability; volunteer to participate in the study. Patients who met the following exclusion criteria were removed from the study: prior physician-diagnosed mental illness before or after breast cancer diagnosis; diagnosed with other cancers; diagnosed with digestive diseases such as enteritis and gastritis; use of antidepressant and anxiolytic medications; use of medications that alter bowel function and metabolism; pregnancy or lactation; use of radiotherapy and chemotherapy medications, antibiotics or probiotics in the past 21 days; other reasons that the researchers thought were not suitable for the study. After receiving written information about the aim of this study, all patients signed their written informed consent before participation. 

### 2.2. Sample Size

According to the previously published literature, the prevalence of depressive symptoms in Chinese breast cancer patients based on the CES-D score was 36.4% [21]. The formula for estimating the sample size was as follows:

$$n=\frac{Z_{\alpha /2}^{2}\times p\times (1-p)}{\delta^{2}}$$

where *α* = 0.05, confidence level = 1 − *α* = 95%, *Z_α/2_* = 1.96, *p* is the prevalence of depressive symptoms, where *p* = 0.364, and *δ* is the permissible error, where *δ* = 0.2 *p* = 0.0728. Therefore, the estimated minimum sample size was 168. 

### 2.3. Depressive Symptoms Assessment

Depressive symptoms were evaluated with the Center for Epidemiologic Studies Depression Scale (CES-D) [22], a self-report questionnaire that contains 20 items with a total score from 0 to 60; the Chinese version has been validated [23,24]. The patients with a CES-D score ≥ 16 were allocated to the depressed breast cancer (DBC) group, while patients with a CES-D score < 16 were in the non-depressed breast cancer (NBC) group.

### 2.4. Anxiety Symptoms Assessment

The Self-Rating Anxiety Scale (SAS) was used to assess the anxiety symptoms of the patients [25]. The SAS consists of 20 items, where each item is scored on a 4-point Likert scale, which is based on the frequency of symptoms, ranging from 1 to 4. The SAS (Chinese version) demonstrated adequate reliability and validity and has been widely used [26]. 

### 2.5. Dietary Intake Assessment and the Calculation of CHEI Component Scores

Three-day twenty-four-hour dietary recall interviews were conducted to evaluate the nutrient intake of breast cancer patients. All the patients were asked to describe in detail the types and amount of food and dietary supplements consumed in the past 24 h, as well as the cooking method. The trained nutrition education research assistants used the food model and atlas to perform the 24 h dietary intake recalls by face-to-face interviews. Dietary data were entered into the Nutrition Calculator v2.7.8.8 (Institute of Nutrition and Food Safety, Chinese Center for Disease Control and Prevention, Beijing, China) to calculate nutrient intake. The average nutrient intake obtained from three 24 h dietary recalls was used as the final data for analysis. 

The Chinese Healthy Eating Index (CHEI), which was established according to the Dietary Guidelines for Chinese (DGC-2016) and whose reliability and validity have been confirmed, was used to evaluate the diet quality [27]. As shown in Table S1, the CHEI scoring system comprises 12 adequacy components (total grains, whole grains and mixed beans, tubers, total vegetables, dark vegetables, fruits, dairy, soybeans, fish and seafood, seeds and nuts, poultry, eggs) and 5 moderation components (red meat, cooking oils, sodium, added sugars, alcohol). CHEI components are scored based on the energy density (as amounts per 1000 calories of intake). Components are scaled from 0 to 5 (0 to 10 for fruits, cooking oils and sodium), with a higher score indicating better diet quality. The total CHEI score is a sum of scores of all 17 components ranging from 0 to 100.

### 2.6. Plasma TRP/LNAA Ratio Determination

Sixty-three blood samples (seventeen from DBC patients and forty-six from NBC patients) were collected in 5 mL tubes containing sodium heparin and centrifuged at 3500 rpm for 10 min. The plasma was stored at −80 °C until analysis. Plasma TRP and LNAA (including valine, Val; isoleucine, Ile; leucine, Lue; tyrosine, Tyr; and phenylalanine, Phe) concentrations were measured by high-performance liquid chromatography (HPLC), and the plasma TRP/LNAA ratio was calculated.

### 2.7. Gut Microbiota Analysis

Fifty-five fecal samples (eighteen from DBC patients and thirty-seven from NBC patients) were collected for 16S rRNA sequencing (Shanghai Majorbio Bio-Pharm Technology Co., Ltd., Shanghai, China). The DNA was extracted using the E.Z.N.A.^®^ soil DNA Kit (Omega Bio-tek, Norcross, GA, USA) according to the manufacturer’s instructions and checked on 1% agarose gel, and the concentration and purity were confirmed with a Nanodrop 2000 UV–Vis spectrophotometer (Thermo Scientific, Wilmington, DE, USA). Purified DNA was further used for PCR amplification of the variable V3–V4 regions of 16S rRNA using primers 338F (5′-ACTCCTACGGGAGGCAGCAG-3′) and 806R (5′-GGACTACHVGGGTWTCTAAT-3′) using an ABI GeneAmp^®^ 9700 PCR thermocycler (ABI, Carlsbad, CA, USA). The process for PCR amplification was as follows: initial denaturation at 95 °C for 3 min, followed by 27 cycles of denaturing at 95 °C for 30 s, annealing at 55 °C for 30 s and extension at 72 °C for 45 s, with a single extension at 72 °C for 10 min, ending at 4 °C. The PCR mixtures contained 5 × TransStart FastPfu buffer 4 μL, 2.5 mM dNTPs 2 μL, forward primer (5 μM) 0.8 μL, reverse primer (5 μM) 0.8 μL, TransStart FastPfu DNA Polymerase 0.4 μL, template DNA 10 ng and, finally, ddH_2_O up to 20 μL. PCR reactions were performed in triplicate. The PCR product was extracted from 2% agarose gel, purified using the AxyPrep DNA Gel Extraction Kit (Axygen Biosciences, Union City, CA, USA) and quantified using a Quantus™ Fluorometer (Promega, Madison, WA, USA). Following the Illumina MiSeq PE300 platform (Illumina, San Diego, CA, USA) 16S Metagenomic Sequencing Library Preparation Program, amplicon multiplexing, merging and sequencing were performed. The raw 16S rRNA gene sequencing reads were demultiplexed, quality filtered by fastp version 0.20.0 and merged by FLASH version 1.2.7. 

Operational taxonomic units (OTUs) with a 97% similarity cutoff were clustered using UPARSE version 7.1, and chimeric sequences were identified and removed. The taxonomy of each OTU representative sequence was analyzed by RDP Classifier version 2.2 against the 16S rRNA database (e.g., Silva v138) using a confidence threshold of 0.7. Alpha diversity was used to analyze the complexity of species diversity in each sample, which involved using QIIME to calculate the Chao (also known as the Chao1 estimator), Shannon and Simpson indexes, and Mothur to assess the rarefaction curve—using the Sobs index (the observed richness) on the OTU level to detect a reasonable amount of sequencing data. When the rarefaction curves for each group tended to be flat, this indicated that the amount of sequencing data was reasonable, and more sequencing data would only produce a small number of new species (such as OTUs). Beta diversity that evaluated differences in the microbial communities was analyzed using a principal coordinate analysis (PCoA) of weighted and unweighted UniFrac distances based on the observed OTUs. The statistical significance was evaluated with an analysis of similarities (ANOSIM). The Wilcoxon rank-sum test was used to detect differences in the alpha diversity of bacterial communities, as well as the differences at the phylum and genus levels (*p*-values were corrected for multiple testing using the Benjamini–Hochberg false discovery rate (FDR) method). To identify the statistically significant biomarkers and the dominant microorganisms in each group, linear discriminatory analysis effect size (LEfSe) was used with default criteria (*p* < 0.05 by a non-parametric factorial Kruskal–Wallis rank-sum test and linear discriminant analysis (LDA) score > 4). The data were analyzed on the Majorbio Cloud Platform (www.majorbio.com, accessed date 28 February 2022).

### 2.8. Statistical Analysis

**Figure 1:**
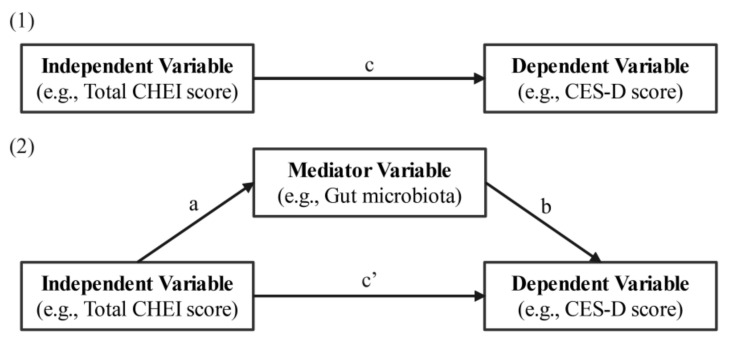
The single-mediator model used to test the association between the nutrients or total CHEI score (i.e., independent variable) and CES-D score (i.e., dependent variable), with the gut microbiota as a mediator: (**1**) Path c represents the simple total effect of the nutrients or total CHEI score on the CES-D score, without adjusting for mediators; (**2**) represents the direct (Path c’) and indirect effects (product of Paths a and b, ab) of the nutrients or total CHEI score on the CES-D score after controlling for the effect of the mediator. CES-D, Center for Epidemiologic Studies Depression; CHEI, Chinese Healthy Eating Index.

The 24 h dietary recall data were entered into the Nutrition Calculator for data processing, and the daily intakes of energy, macronutrients, micronutrients and various food components were calculated. Data analyses were performed using SPSS 22.0 (SPSS Inc., Chicago, IL, USA). Continuous variables were expressed as mean ± SD, and categorical variables were presented as frequencies (N) and percentages (%). The Kolmogorov–Smirnov test or a Q–Q plot was used to evaluate the normal distribution. A chi-square test or a chi-square test with continuity correction was used to analyze the differences between categorical variables. An independent sample *t*-test and the Mann–Whitney U test were used to assess the differences in normally and non-normally distributed data between the DBC and NBC groups, respectively. Pearson or Spearman correlation analysis was used to examine the correlations between nutrient intake or CHEI component scores and the CES-D score, as well as correlations between dietary variables and bacterial taxa. Simple linear regression was used to assess the associations between the gut microbiota (alpha diversity, beta diversity, phyla) and the CES-D score, as well as the dietary intake and gut microbiota. To determine whether the dietary intake was associated with depression (CES-D score), or whether the association was mediated by the gut microbiota (alpha diversity), a simple mediation analysis was conducted using the PROCESS macro (v4.0 by Andrew Hayes) for SPSS (Figure 1). A bootstrap method using iterations of computed samples (5000 replications) was adopted to determine the significance of the indirect effects (ab) of the gut microbiota (alpha diversity) for the association of nutrients, the total CHEI score and the CES-D score. If the 95% confidence interval (CI) did not include 0, this meant that the mediation effect was significant. Age, BMI, family monthly income, education level, menopausal status, marital status, employment, residence and SAS score were included as covariates in the models. A value of *p* < 0.05 was considered statistically significant. 

## 3. Results

### 3.1. Patient Characteristics

Out of the 205 patients, 60 (29.3%) patients were allocated to the DBC group by the CES-D score, and 145 (70.7%) patients were in the NBC group. Demographic and clinical characteristics, blood routine and blood biochemical indexes and scores of questionnaires of the two groups are presented in Table S2. Statistical differences were found in age (*p* = 0.018), menopausal status (*p* = 0.016), employment (*p* < 0.001), serum globulin (*p* = 0.046), CES-D score (*p* < 0.001) and SAS score (*p* < 0.001) between the two groups.

### 3.2. Nutrient Intakes, CHEI Component Scores and Plasma Amino Acid Contents of the Patients

The nutrient intakes of the depressed and non-depressed breast cancer patients are shown in Table 1. Compared with DBC patients, the energy, protein, dietary fiber, vitamin A, vitamin B2, niacin, calcium, phosphorus, potassium, iron, zinc, selenium, manganese and tryptophan intake, total CHEI score and fruit score of the NBC patients were all remarkably higher (*p* < 0.05), which were all negatively correlated with the CES-D score (*p* < 0.05, Table S3). The plasma amino acid contents of the depressed and non-depressed breast cancer patients are presented in Table S4. Tryptophan levels of the NBC patients were remarkably higher than those of the DBC patients (*p* = 0.047). No significance was found for tyrosine, valine, phenylalanine, isoleucine, leucine and TRP/LNAAs between the two groups (*p* > 0.05).

### 3.3. Gut Microbiota Diversity of the Patients

**Figure 2:**
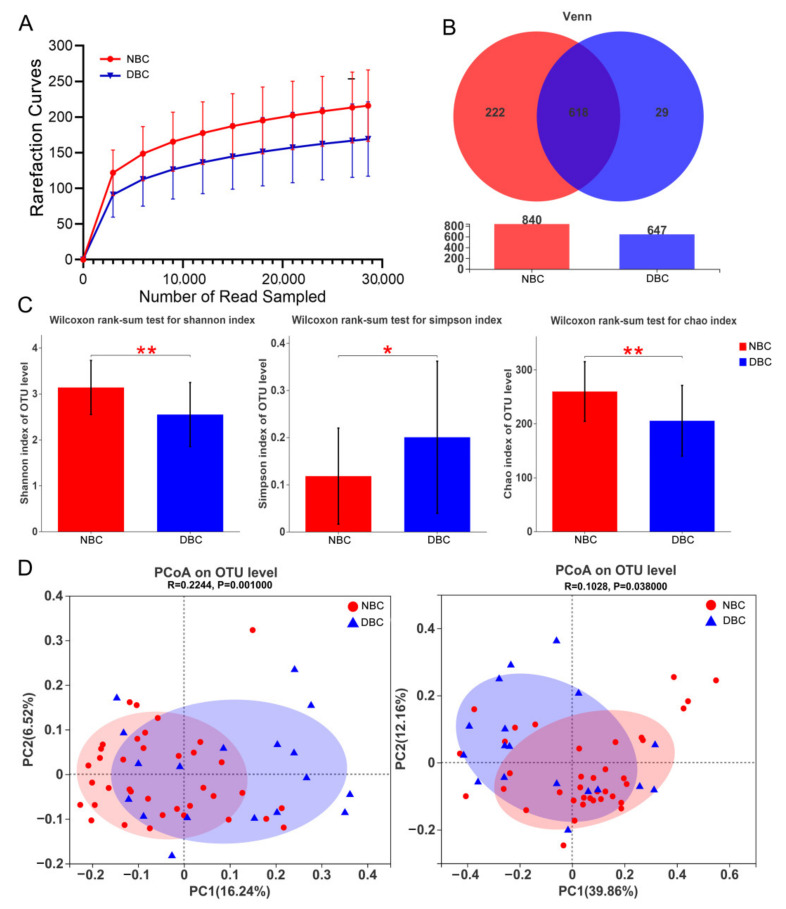
Differences in gut microbiota structure between the DBC and NBC patients. (**A**) The rarefaction curves to assess the sequencing depth. (**B**) Venn diagram showing the shared number of operational taxonomic units by DBC and NBC patients. (**C**) Shannon index, Simpson index and Chao index were used to measure richness and diversity. A higher value of the Shannon index indicates a higher community diversity, whereas a higher Simpson index indicates a lower community diversity. The Wilcoxon rank-sum test was used. (**D**) Unweighted (left) and weighted (right) UniFrac distance-based principal coordinate analysis (PCoA). The percentages of variation explained by PC1 and PC2 are shown on the axis. Distances between the samples were based on similarity in OTU composition (OTU similarity: 97%). A greater distance indicates a lower similarity, and similar OTUs cluster together. The statistical significance was evaluated with analysis of similarities (ANOSIM). DBC, depressed breast cancer patients (*n* = 18); NBC, non-depressed breast cancer patients (*n* = 37). * *p* < 0.05, ** *p* < 0.01.

To evaluate the sequencing data, we performed rarefaction curve analysis with 97% similarity in terms of OTUs. As shown in Figure 2A, the rarefaction results indicate that our sequencing depth was sufficient to study the microbial diversity of each sample. According to the Venn diagram, 618 OTUs were shared between the DBC and NBC patients (Figure 2B). Alpha diversity analysis showed the richness, diversity and evenness between the two groups. The richness (Chao index) and diversity (Shannon index) of the DBC patients were both significantly lower, while the Simpson index was significantly higher than that of the NBC patients (Figure 2C), indicating a significant decrease in the diversity of the gut microbiota in the DBC patients. A higher Shannon index indicates a higher community diversity, whereas a higher Simpson index indicates a lower community diversity. The PCoA (Figure 2D) results show that the gut microbiota of the DBC patients was significantly different from that of the NBC patients in both unweighted (left) UniFrac distances (ANOSIM R = 0.224, *p* = 0.001) and weighted (right) UniFrac distances (ANOSIM R = 0.103, *p* = 0.038). 

### 3.4. Gut Microbiota Composition of the Patients 

**Figure 3:**
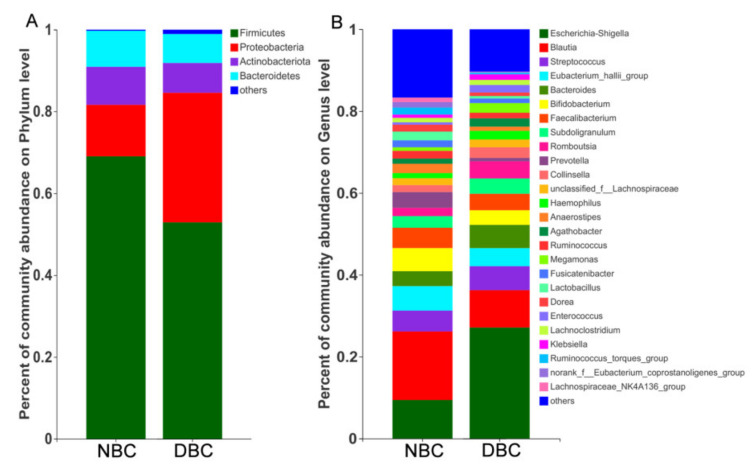
Gut microbiota composition in depressed and non-depressed breast cancer patients. (**A**) The community structures of different microbes at the phylum level. (**B**) The community structures of different microbes at the genus level. DBC, depressed breast cancer patients (*n* = 18); NBC, non-depressed breast cancer patients (*n* = 37).

In both groups, Firmicutes was the highest dominant phylum, and the abundance of such a phylum was 69.1% and 52.9% in the NBC and DBC patients, respectively (Figure 3A). The second major phylum was Proteobacteria, and the abundance of such a phylum was 31.7% and 12.6% in the NBC and DBC patients, respectively. The relative abundance of Firmicutes in the NBC patients was significantly higher (*p* = 0.028), while the relative abundance of Proteobacteria was remarkably lower than that of the DBC patients (*p* = 0.011, Table S5). However, after the results of the Wilcoxon rank-sum test were corrected with FDR correction, there was no statistical difference at the phylum level between the NBC and DBC patients. The dominant genera (>5%) in the gut microbiota were *Escherichia-Shigella*, *Blautia* and *Streptococcus* in the two groups; in addition, *Eubacterium_hallii_group* and *Bifidobacterium* were only dominant in the NBC patients, while *Bacteroides* was only dominant in the DBC patients (Figure 3B). In terms of the genera, the relative abundances of *Blautia* (*p* = 0.027) and *Anaerostipes* (*p* = 0.044) in the DBC patients were significantly lower, while the relative abundance of *Escherichia-Shigella* (*p* = 0.010) was significantly higher than that in the NBC patients (Table S6). The relative abundances of *norank_f_Ruminococcaceae* (corrected *p*-value = 0.049) and *norank_f_Oscillospiraceae* (corrected *p*-value = 0.049) of the NBC patients were both statistically higher than those of the DBC patients after FDR correction. 

**Figure 4:**
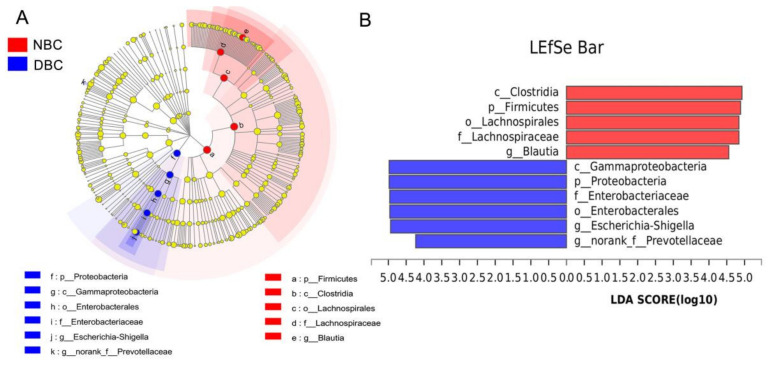
Differentiated microbes between depressed and non-depressed breast cancer patients. (**A**) LEfSe analysis was used to distinguish the differential microbes between the DBC and NBC patients. The different colored nodes represent microbial populations that were significantly enriched in the corresponding groups and that showed significant differences between the groups. (**B**) LDA was performed, and only the microbiota with LDA scores of > 4 are shown. DBC, depressed breast cancer patients (*n* = 18); NBC, non-depressed breast cancer patients (*n* = 37).

We used LEfSe to identify specific bacterial taxa significantly differentiated between the two groups (Figure 4). There were 11 differentiated taxa (from phylum to genus) found with an LDA score > 4.0, among which c_Clostridia (*p* = 0.034), p_Firmicutes (*p* = 0.027), o_Lachnospirales (*p* = 0.024), f_Lachnospiraceae (*p* = 0.024) and *g_Blautia* (*p* = 0.026) were more abundant in the NBC patients, whereas c_Gammaproteobacteria (*p* = 0.011)*,* p_Proteobacteria (*p* = 0.011), f_Enterobacteriaceae (*p* = 0.008), o_Enterobacterales (*p* = 0.008), *g_Escherichia-Shigella* (*p* = 0.010) and *g_norank_f_Prevotellaceae* (*p* = 0.041) were more abundant in the DBC patients.

### 3.5. Associations of Gut Microbiota with CES-D Score and Dietary Intake 

**Figure 5:**
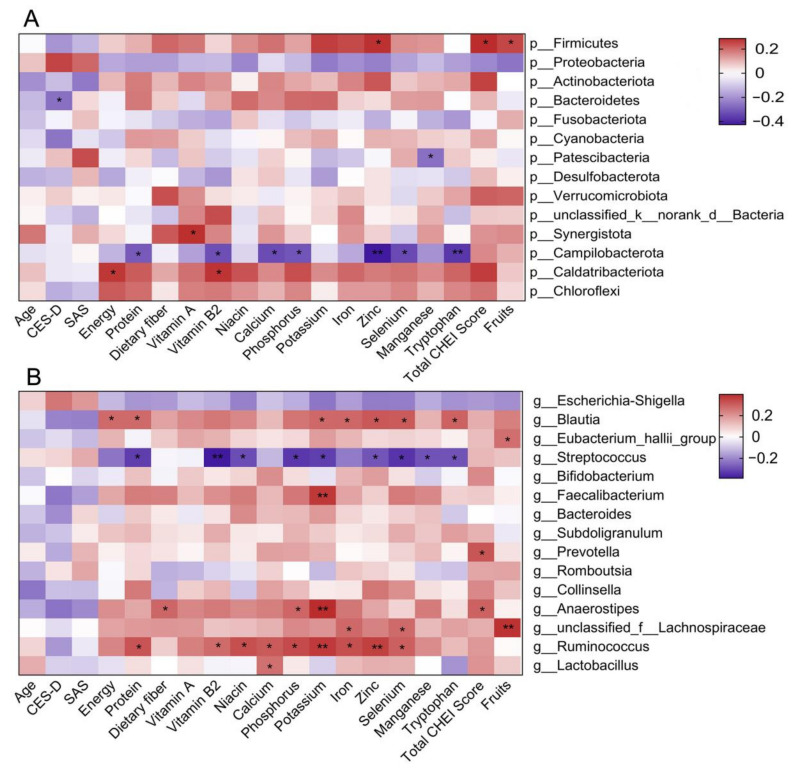
Spearman and Pearson correlation analysis between the abundance of the top 15 taxa at the phylum (**A**) and genus (**B**) levels and the CES-D score, nutrient intakes and CHEI component scores (*n* = 55). The color denotes the strength of the correlation, where red indicates positive, and blue indicates negative. The correlations between Firmicutes, except for vitamin B2, manganese and the CHEI fruit score, were performed with Pearson correlation analysis; the rest of the associations were analyzed with Spearman correlation analysis. CES-D, Center for Epidemiological Studies-Depression; SAS, Self-Rating Anxiety Scale; CHEI, Chinese Healthy Eating Index. * *p* < 0.05, ** *p* < 0.01.

The associations between the relative abundances of the top 15 taxa at the genus and phylum levels and the CES-D score, nutrient intakes and CHEI component scores are shown in Figure 5. The relative abundance of Bacteroidetes had a significantly negative correlation with the CES-D score. The relative abundance of Firmicutes had significantly positive correlations with dietary zinc intake, the total CHEI score and the fruit score, while the relative abundance of Campilobacterota had significantly negative associations with the dietary intake of protein, vitamin B2, calcium, phosphorus, zinc, selenium and tryptophan. The relative abundance of *Blautia* was positively correlated with the dietary intake of energy, protein, potassium, iron, zinc, selenium and tryptophan. However, the relative abundance of *Streptococcus* was negatively correlated with the dietary intake of protein, vitamin B2, niacin, phosphorus, potassium, zinc, selenium, manganese and tryptophan.

### 3.6. Associations between Diet and Depression with Gut Microbiota as a Mediator

The correlation analysis between nutrient intakes, CHEI component scores and the CES-D score from the 55 patients with 16S rRNA sequencing data is presented in Table 2. Dietary protein, dietary fiber, vitamin A, vitamin B2, niacin, calcium, phosphorus, potassium, iron, zinc, selenium, manganese and the total CHEI score all significantly correlated with the CES-D score (*p* < 0.05). The simple linear regression model showed that the CES-D score was significantly correlated with the Chao index (β = −0.348, *p* < 0.001), Shannon index (β = −0.334, *p* < 0.001) and Simpson index (β = 0.260, *p* = 0.006) (Table 3). The total CHEI score was significantly correlated with the Chao index (β = 0.387, *p* = 0.014), Shannon index (β = 0.369, *p* = 0.018) and Simpson index (β = −0.426, *p* = 0.008, Table S7). The intakes of calcium (β = 0.305, *p* = 0.048) and phosphorus (β = 0.313, *p* = 0.044) were positively associated with the Chao index. Furthermore, the intakes of calcium (β = 0.316, *p* = 0.038) and selenium (β = 0.347, *p* = 0.022) both had a positive association with the Shannon index. We conducted a mediation analysis to understand whether diet-induced differences in the gut microbiota induced depression (Table 4). The results show that the association between the total CHEI score and the CES-D score arose through alpha diversity suggested by the Chao index (ab = −0.119; 95% CI = −0.249, −0.004) and Simpson index (ab = −0.090, 95% CI = −0.200, −0.008). Calcium, phosphorus and selenium intakes were associated (total effect (c)) with the CES-D score. A higher CES-D score was associated with lower calcium (c = −0.006, 95% CI = −0.011, −0.0004), phosphorus (c = −0.006, 95% CI = −0.011, −0.0007) and selenium (c = −0.086, 95% CI = −0.149, −0.023) intakes, indicating that these minerals’ intakes were negatively associated with depression, but not through the gut microbiota.

## 4. Discussion

This cross-sectional study investigated the relationships among diet, the gut microbiota and depressive symptoms in breast cancer patients. Our results indicate that depressive symptoms in breast cancer patients might be partially associated with the inadequate intake of certain nutrients and fruits. Moreover, non-depressed breast cancer patients demonstrated greater gut microbiota diversity and a healthier composition. An association between diet quality and depressive symptoms was also observed: a higher total CHEI score was inversely associated with the CES-D score, and alpha diversity was a significant mediator of the association between diet quality and depressive symptoms. Calcium, phosphorus and selenium affected depressive symptoms independent of the gut microbiota. 

Comorbid depression is common in breast cancer patients and associated with a decreased life quality and reduced compliance with treatment [2,28]. We found that 29.3% of breast cancer patients had depressive symptoms evaluated with the CES-D score, which was lower than the prevalence of depression in previous studies in China [21,29]. Younger pre-menopausal women were more likely to suffer from depressive symptoms than older post-menopausal women, which might be attributed to the notion that younger patients might pay more attention to their own image and worry about their disease affecting marriage, family stability, fertility and their future career [30]. We also observed that employed women with breast cancer were particularly prone to depressive symptoms. In employed breast cancer patients, adverse reactions to breast cancer treatment could negatively impact survivors’ ability to work [31], and they were afraid of not only being discriminated against by their colleagues [32], but also losing their job, which possibly led to a higher rate of depression. Therefore, clinical staff should be concerned about the depressive symptoms of young breast cancer patients who are still working and offer reasonable suggestions to deal with depressive symptoms.

The relationship between diet and depression has long been a topic of interest. Dietary modification may influence a great many factors that control the development and trajectory of depression [7], although the mechanisms of these interactions are not completely understood. Both cross-sectional and longitudinal studies have revealed that a Western diet or a highly processed diet was associated with a greater risk of depression [11,33]. Conversely, adherence to Mediterranean dietary patterns was inversely associated with the odds of depression [34]. Many previous studies have focused on the relationship between dietary patterns and depression. In this study, we calculated the nutrient intakes and their associations with depression, which provided a more accurate method for the prevention or treatment of depressive symptoms. A randomized controlled trial demonstrated a 3-month dietary intervention had a fairly significant effect on moderate to severe depression, with a remarkably greater improvement in the dietary intervention group and 32% of patients in this group achieving remission [35]. Due to the universality of food as a modifiable risk factor, even minor improvements in the diet can translate to considerable benefits for depression. We found that compared with depressed breast cancer patients, non-depressed patients showed higher nutrient intakes, including energy, protein, dietary fiber, vitamin A, vitamin B2, niacin, calcium, phosphorus, potassium, iron, zinc, selenium, manganese and tryptophan, and these nutrient intakes were all negatively correlated with the CES-D score. In another study, parts of these nutrients were also related to depression, including ω-3 fatty acids, antioxidants (vitamin C and zinc), vitamin B12, folic acid and magnesium [36], indicating that the appropriate nutrient intake is critical to counteract depressive symptoms. Therefore, it is better for the depressed patients to increase their animal-derived food intake to obtain enough high-quality protein, and also improve the intake of vegetables and fruits to obtain sufficient vitamins and minerals as well.

Recent data have highlighted the contribution of diet quality to the development of depressive symptoms. In a national cross-sectional study in Spain, non-depressive people had a higher diet quality than depressed people [9]. In a prospective investigation, increasing HEI-Canada scores were associated with fewer physician visits for depression in adults living in Alberta [37]. Compared to those with the best diet quality, individuals with the worst diet quality were 39% more likely to suffer from major depressive episodes [38]. We found that the total CHEI score and the fruit score of non-depressed breast cancer patients were both higher than those of depressed patients, which is consistent with previous findings that low HEI and fruit scores were associated with depression [39]. Diet quality was also an essential factor influencing the manifestation of depressive symptoms in breast cancer survivors [40]. Consequently, in addition to increasing the intake of balanced nutrients in breast cancer patients, dietary interventions may also improve depressive symptoms by enhancing the diet quality and consumption of healthy food, such as fruits. 

Tryptophan is a so-called natural alternative to traditional antidepressants, and a tryptophan-rich diet is a potentially protective factor for depression. It is considered that the plasma concentration of branched-chain amino acids (BCAAs), in competition with free tryptophan, governs the rate of entry of tryptophan into the brain [41]. The ingestion of BCAAs lowered brain tryptophan uptake and 5-HT synthesis [42]. A positive correlation between the concentration of BCAAs and the severity of major depressive disorder (MDD) was revealed, and the plasma BCAAs decreased after MDD patients received antidepressant treatment [43]. We also observed that depressed breast cancer patients had a lower intake of tryptophan and decreased plasma tryptophan levels compared to the non-depressed patients, without a significant difference in LNAAs and the ratio of Trp/LNAAs. We speculated that this might be attributed to the limited plasma samples from breast cancer patients; thus, more samples are needed for further analysis. 

Microbiota–gut–brain communication has been shown to play a critical role in depression. Since bidirectional communication between the gut microbiota and the brain could affect neurotransmission and behavior, the modulation of the gut microbiota could provide a new therapy for depression intervention [44]. Although previous studies suggested that no significant difference existed in the microbial diversity of depression patients compared to the healthy group [45], we found that non-depressed patients demonstrated greater gut microbiota diversity and an enriched gut microbiota composition compared to depressed patients, which is in line with the findings of significant differences in the Chao index and Shannon index between depressed and non-depressed participants [46]. As one of the dominant phyla, Proteobacteria were increased in depressed patients compared with healthy controls, accompanied by decreased Firmicutes [47]. Our results also show that depressed breast cancer patients had an increased relative abundance of Proteobacteria and a lower Firmicutes abundance than non-depressed patients, suggesting that the overrepresentation of Proteobacteria and lower Firmicutes abundance might be associated with depression. Although studies that focused on the effects of the *Blautia* genus on psychiatric disorders illustrated conflicting results, one consistent finding was that the gut microbial neurotransmitter γ-aminobutyric acid (GABA), a downstream product of *Blautia*-dependent arginine metabolism, was related to a decreased risk of Alzheimer’s disease. In the same study, the association between *Blautia* and MDD showed a similar trend [48]. *Escherichia-Shigella* belongs to the *Enterobacteriaceae* family, whose overrepresentation has been proved to cause gut inflammation and increased gut permeability [49] and was positively associated with DASS-42-Depression scores [39]. We observed that *Escherichia-Shigella* was overrepresented in the depressed patients, while *Blautia* was decreased compared with non-depressed patients. The relative abundances of *norank_f__Ruminococcaceae* and *norank_f__Oscillospiraceae* in the NBC patients were significantly higher than those in the DBC patients. Until now, we knew little about the genera of *norank_f_Ruminococcaceae* and *norank_f__Oscillospiraceae*. Further study should be conducted to identify the function of these microbes. Food intake or adherence to an entire dietary pattern (in a healthy or unhealthy direction) led to alterations in the gut microbiota [50]. Certain components from fruits and vegetables (such as polyphenols) might affect the gut microbiota by acting as prebiotics [51]. Mediation analysis revealed that diet quality evaluated by the total CHEI score regulated depressive symptoms through affecting gut microbiota diversity. Moreover, calcium, phosphorus and selenium intakes were negatively associated with the CES-D score in breast cancer patients independent of the gut microbiota. These findings suggest that diet might regulate depression via gut microbiota-dependent and independent pathways. Given the close connection between depression and the gut microbiota, future causal studies of the relationship between the above microbes and depression are warranted.

To date, this is the first study to investigate the associations among diet, the gut microbiota and depression in breast cancer patients. However, there are some potential limitations in this research that should be considered. First, although the CES-D scale is easy and quick to apply and widely used in epidemiological studies, it is a screening scale, not a diagnostic tool. Additionally, patients diagnosed with depression before or after their cancer diagnosis were excluded from the study, and we only focused on the mild symptomatology of depression. Second, this study was carried out in a single city; thus, a multicenter, population-based study should be conducted in the future. Third, due to the significant impact of chemotherapy on diet and the gut microbiota, blood and fecal samples were collected before the initiation of chemotherapy. We only had 55 fecal samples and 63 blood samples. Finally, this study is only a cross-sectional design, and longitudinal studies are needed to clarify the trend of depression in breast cancer patients and the role of diet in depression development.

## 5. Conclusions

This study found that the incidence of depressive symptoms in breast cancer patients was 29.3%. Compared to non-depressed patients, the depressed patients had lower nutrient intakes, a poor diet quality and gut dysbiosis. Diet quality regulated depression via altering gut microbiota diversity, while calcium, phosphorus and selenium affected depression independent of the gut microbiota. With continued research investigating these associations among diet, the gut microbiota and depression, we will further our understanding and make advances in obtaining a well-understood and well-guided holistic approach to treating and preventing depressive symptoms in breast cancer patients.

## Figures and Tables

**Table 1:** Nutrient intakes and CHEI component scores of the depressed and non-depressed breast cancer patients.

	NBC (*n* = 145)	DBC (*n* = 60)	*t*/Z	*p*-Value
**Nutrients**				
Energy (kcal/d) ^1^	1539.29 ± 428.12	1403.40 ± 273.64	2.569	0.011
Protein (g/d) ^1^	75.24 ± 28.78	65.45 ± 16.64	2.891	0.004
Fat (g/d) ^1^	57.78 ± 23.83	54.21 ± 17.43	0.982	0.327
Carbohydrate (g/d) ^1^	174.75 ± 63.53	165.27 ± 59.86	0.929	0.354
Dietary fiber (g/d) ^1^	12.52 ± 6.01	10.38 ± 6.26	2.162	0.032
Cholesterol (g/d) ^2^	617.89 ± 348.54	541.54 ± 282.61	−0.912	0.362
Vitamin A (μgRAE/d) ^2^	530.78 ± 290.36	378.49 ± 209.22	−3.340	0.001
Vitamin D (μg/d) ^2^	11.92 ± 25.71	9.38 ± 19.92	−0.935	0.350
Vitamin E (mg/d) ^1^	19.73 ± 11.53	17.06 ± 8.00	1.792	0.075
Vitamin B1 (mg/d) ^1^	0.88 ± 0.39	0.77 ± 0.34	1.855	0.065
Vitamin B2 (mg/d) ^2^	1.26 ± 0.66	0.99 ± 0.46	−3.060	0.002
Vitamin B6 (mg/d) ^2^	0.24 ± 0.20	0.20 ± 0.13	−0.408	0.683
Vitamin C (mg/d) ^2^	132.56 ± 85.26	119.36 ± 86.31	−1.221	0.222
Folate (μg/d) ^1^	154.05 ± 82.75	133.80 ± 78.43	1.479	0.141
Niacin (mg/d) ^2^	15.00 ± 6.22	12.31 ± 4.20	−2.756	0.006
Calcium (mg/d) ^1^	621.73 ± 283.03	531.73 ± 267.13	1.978	0.049
Phosphorus (mg/d) ^1^	1023.22 ± 321.55	877.03 ± 249.03	3.299	0.001
Potassium (mg/d) ^1^	2164.18 ± 763.69	1842.64 ± 760.09	2.583	0.011
Sodium (mg/d) ^1^	2692.12 ± 463.39	2699.23 ± 517.62	−0.090	0.928
Magnesium (mg/d) ^2^	301.73 ± 121.62	260.12 ± 104.34	−1.850	0.064
Iron (mg/d) ^2^	19.38 ± 8.23	15.91 ± 5.31	−2.598	0.009
Iodine (μg/d) ^2^	23.65 ± 15.83	19.71 ± 14.46	−1.724	0.085
Zinc (mg/d) ^1^	10.39 ± 4.17	8.83 ± 3.05	2.810	0.006
Selenium (μg/d) ^1^	55.43 ± 23.85	45.77 ± 20.41	2.557	0.011
Copper (mg/d) ^2^	2.08 ± 1.67	1.85 ± 1.59	−0.986	0.324
Manganese (mg/d) ^2^	3.98 ± 1.98	3.23 ± 1.30	3.007	0.003
Choline (mg/d) ^2^	30.31 ± 22.62	35.57 ± 29.12	−0.730	0.465
Biotin (μg/d) ^2^	5.11 ± 5.02	5.22 ± 4.77	−0.062	0.951
Tryptophan (mg/d) ^1^	659.99 ± 201.87	579.41 ± 156.87	2.501	0.013
SFA (g/d) ^1^	13.28 ± 6.47	11.89 ± 5.22	1.345	0.180
MUFA (g/d) ^1^	16.53 ± 7.50	15.15 ± 5.71	1.310	0.193
PUFA (g/d) ^1^	10.96 ± 4.88	10.07 ± 4.17	1.109	0.269
**CHEI component scores**				
Total CHEI score ^1^	67.40 ± 6.98	64.80 ± 7.26	2.195	0.029
Total grains ^2^	3.69 ± 1.23	3.58 ± 1.25	−0.583	0.560
Whole grains and mixed beans ^2^	1.49 ± 1.76	1.38 ± 1.45	−0.797	0.425
Tubers ^2^	1.74 ± 2.18	1.57 ± 1.87	−0.126	0.900
Total vegetables ^2^	3.60 ± 1.49	3.58 ± 1.24	−0.420	0.675
Dark vegetables ^2^	3.93 ± 1.46	3.97 ± 1.41	−0.186	0.853
Fruits ^2^	9.43 ± 1.82	8.46 ± 2.80	−2.275	0.023
Eggs ^2^	3.92 ± 1.66	3.63 ± 1.99	−0.601	0.548
Soybeans ^2^	1.41 ± 2.15	1.63 ± 2.22	−0.780	0.435
Dairy ^2^	2.88 ± 2.20	2.67 ± 2.07	−0.740	0.459
Seed and nuts ^2^	1.53 ± 2.06	1.32 ± 2.12	−1.096	0.273
Fish and seafood ^2^	3.19 ± 2.18	3.43 ± 2.13	−0.869	0.385
Poultry ^2^	1.26 ± 2.17	1.08 ± 2.05	−0.481	0.631
Red meat ^2^	3.12 ± 1.81	2.84 ± 1.98	−0.691	0.490
Added sugars ^2^	4.81 ± 0.82	4.87 ± 0.73	−0.266	0.790
Cooking oils ^2^	9.86 ± 0.51	9.73 ± 0.81	−1.059	0.289
Alcohol ^2^	5.00 ± 0.00	5.00 ± 0.00	0.000	1.000
Sodium ^2^	6.53 ± 2.10	6.03 ± 1.78	−1.863	0.062

Data are shown as mean and standard deviation (SD) for continuous variables. ^1^ Independent sample *t*-test; ^2^ Mann–Whitney test; DBC, depressed breast cancer patients; NBC, non-depressed breast cancer patients; SFA, saturated fatty acids; MUFA, monounsaturated fatty acids; PUFA, polyunsaturated fatty acids; CHEI, Chinese Healthy Eating Index.

**Table 2:** Correlation between the CES-D score and nutrient intakes and CHEI component scores (*n* = 55).

Variables	r	*p*-Value	Variables	r	*p*-Value
Energy ^1^	−0.219	0.108	Potassium ^1^	−0.461	<0.001
Protein ^1^	−0.347	0.009	Iron ^1^	−0.349	0.009
Dietary fiber ^1^	−0.331	0.014	Zinc ^1^	−0.371	0.005
Vitamin A ^1^	−0.274	0.043	Selenium ^1^	−0.389	0.003
Vitamin B2 ^2^	−0.276	0.042	Manganese ^2^	−0.292	0.031
Niacin ^1^	−0.321	0.017	Tryptophan ^1^	−0.233	0.091
Calcium ^1^	−0.353	0.008	Total CHEI score ^1^	−0.402	0.002
Phosphorus ^1^	−0.447	0.001	Fruits ^2^	−0.002	0.991

CES-D, Center for Epidemiologic Studies Depression; CHEI, Chinese Healthy Eating Index. ^1^ Pearson correlation analysis; ^2^ Spearman correlation analysis.

**Table 3:** Linear regression analyses of the associations between the gut microbiota and the CES-D score (*n* = 55).

Variables	CES-D Score			
	R^2^	β	95%CI	*p*-Value
Chao index	0.721	−0.348	−0.061, −0.021	<0.001
Shannon index	0.710	−0.334	−5.619, −1.709	<0.001
Simpson index	0.678	0.260	4.610, 25.499	0.006
PC1	0.639	−0.175	−10.898, 1.020	0.102
Bacteroidetes	0.637	−0.153	−0.198, 0.024	0.121

Simple linear regression models were used after adjusting for age, BMI, family monthly income, education level, menopausal status, marital status, employment, residence and SAS score. CES-D, Center for Epidemiologic Studies Depression.

**Table 4:** Total, direct and indirect effects of diet on the CES-D score with the gut microbiota as a mediator in breast cancer participants (*n* = 55).

Variables	Total Effect (c)			Direct Effect (c’)			Indirect Effect (ab)		
Independent Variable and Mediators	β	SE	CI	β	SE	CI	β	SE	CI
**Total CHEI score**	−0.228	0.102	−0.433, −0.023						
via Chao				−0.109	0.098	−0.306, 0.088	−0.119	0.063	−0.249, −0.004
via Shannon				−0.121	0.099	−0.320, 0.079	−0.108	0.061	−0.230, 0.004
via Simpson				−0.139	0.106	−0.352, 0.074	−0.090	0.048	−0.200, −0.008
**Calcium**	−0.006	0.003	−0.011, −0.0004						
via Chao				−0.003	0.002	−0.008, 0.002	−0.003	0.002	−0.006, 0.0001
via Shannon				−0.003	0.003	−0.008, 0.002	−0.002	0.002	−0.006, 0.0001
**Phosphorus**	−0.006	0.002	−0.011, −0.0007						
via Chao				−0.003	0.002	−0.008, 0.001	−0.002	0.002	−0.006, 0.0002
**Selenium**	−0.086	0.031	−0.149, −0.023						
via Shannon				−0.055	0.031	−0.116, 0.007	−0.031	0.017	−0.063, 0.003

Mediation analyses were conducted through linear regression using the PROCESS macro for SPSS adjusting for age, BMI, family monthly income, education level, menopausal status, employment, residence, marital status and SAS score. A bootstrap method using iterations of computed samples (5000) was used to determine the significance of the indirect effects.

## Data Availability

The data presented in this study are available on request from the corresponding author.

## Supplementary Materials

The following supporting information can be downloaded at: https://www.mdpi.com/article/10.3390/nu14061186/s1, Table S1: Chinese healthy eating index components and standard for scoring; Table S2: Characteristics of the non-depressed and depressed breast cancer patients; Table S3: Correlation between CES-D score and nutrient intakes, CHEI component score (*n* = 205); Table S4: Plasma amino acids of the depressed and non-depressed breast cancer patients; Table S5: Differences of gut microbiota composition between the depressed and non-depressed breast cancer patients at the phylum level. Table S6. Differences of gut microbiota composition between the depressed and non-depressed breast cancer patients at the genus level. Table S7: Linear regression analyses of the associations between diet and gut microbiota (*n* = 55).
