# Supplementary material for: Impact of Gut Microbiota on the Association between Diet and Depressive Symptoms in Breast Cancer
Source: Nutrients. 2022 Mar 11;14(6):1186. doi: 10.3390/nu14061186 (PMC8948907; doi:10.3390/nu14061186)
Supplement: Supplementary file 1 [file nutrients-14-01186-s001.zip › nutrients-1595902-supplementary.pdf]

Table S1. Chinese healthy eating index components and standard for scoring

Component	Score			
	0		5	10
**Adequacy**				
Total grains	0	→	≧2.5SP/1000kcal	
Whole grains and mixed beans	0	→	≧0.6SP/1000kcal	
Tubers	0	→	≧0.3SP/1000kcal	
Total vegetables	0	→	≧1.9SP/1000kcal	
Dark vegetables	0	→	≧0.9SP/1000kcal	
Fruits	0	→		≧1.1SP/1000kcal
Dairy	0	→	≧0.5SP/1000kcal	
Soybeans	0	→	≧0.4SP/1000kcal	
Fish and seafood	0	→	≧0.6SP/1000kcal	
Poultry	0	→	≧0.3SP/1000kcal	
Eggs	0	→	≧0.5SP/1000kcal	
Seeds and nuts	0	→	≧0.4SP/1000kcal	
**Limitation**				
Red meat	≧3.5	→	≦0.4SP/1000kcal	
Cooking oils	≧32.6	→		≦15.6g/1000kcal
Sodium	≧3608	→		≦1000mg/1000kcal
Added sugars	≧20%	→	≦10% of energy	
Alcohol	≧25g (men) /15g (women)	→	≦60g(men)/40g(women)	

Table S2. Characteristics of the non-depressed and depressed breast cancer patients

	NBC (n = 145)	DBC (n = 60)	t /Z/χ^2	*p*-Value
**Demographic characteristics**				
Age (years) [1]	54.0 ± 9.4	50.2 ± 10.5	2.387	**0.018**
BMI [1]	23.6 ± 3.3	23.5 ± 3.0	0.287	0.775
Family monthly income (RMB) [2]				
<2000	30 (20.7%)	12 (20.0%)		
2000～5000	88 (60.7%)	43 (71.7%)	3.687	0.158
＞5000	27 (18.6%)	5 (8.3%)		
Education level [2]				
Primary school and below	33 (22.8%)	13 (21.7%)		
Middle school	59 (40.7%)	23 (38.3%)	2.162	0.539
High school or secondary school	31 (21.4%)	10 (16.7%)		
Junior college or above	22 (15.2%)	14 (23.3%)		
Menopausal status [2]				
Pre-menopausal	51 (35.2%)	32 (53.3%)	5.809	**0.016**
Post-menopausal	94 (64.8%)	28 (46.7%)		
Marital status [3]				
Married	134 (92.4%)	56 (93.3%)	0.000	1.000
Widowed/divorced/separated/single	11 (7.6%)	4 (6.7%)		
Employment [2]				
Employed	38 (26.2%)	34 (56.7%)	17.818	**<0.001**

Unemployed	32 (22.1%)	10 (16.7%)		
Retired	75 (51.7%)	16 (26.7%)		
Residence [2]				
Urban areas	64 (44.1%)	22 (36.7%)		
Towns	42 (29.0%)	25 (41.7%)	3.117	0.210
Rural areas	39 (26.9%)	13 (21.7%)		
**Clinical characteristics**				
Cancer stage [2]				
I	49 (33.8%)	17 (28.3%)		
II	67 (46.2%)	29 (48.3%)	0.659	0.719
III	29 (20.0%)	14 (23.3%)		
Type of surgery [2]				
Simple mastectomy	84 (57.9%)	31 (51.7%)		
Modified radical mastectomy	41 (28.3%)	18 (30.0%)	0.919	0.632
Breast-conserving surgery	20 (13.8%)	11 (18.3%)		
**Blood routine and blood biochemical indexes**				
Red blood cell ($\times 10^{12}$/L) [1]	3.95 ± 0.41	3.95 ± 0.36	0.026	0.979
White blood cell ($\times 10^{9}$/L) [4]	5.10 ± 2.09	5.56 ± 2.27	−1.067	0.286
Platelet ($\times 10^{9}$/L) [1]	245.88 ± 68.97	244.29 ± 60.05	0.138	0.890
Hemoglobin (g/L) [1]	122.04 ± 12.43	119.92 ± 10.89	1.020	0.309
Total lymphocyte count ($\times 10^{9}$/L) [4]	1.26 ± 0.37	1.29 ± 0.64	−1.028	0.304
Neutrophil ($\times 10^{9}$/L) [4]	3.36 ± 1.95	3.57 ± 1.55	−0.966	0.334
Hypersensitive C-reactive protein (mg/L) [4]	3.50 ± 3.02	5.15 ± 5.77	−0.895	0.371

Serum albumin (g/L) [1]	43.52 ± 3.03	43.14 ± 3.52	0.691	0.491
Serum globulin (g/L) [1]	28.17 ± 3.28	26.95 ± 3.91	2.008	**0.046**
Serum total protein (g/L) [1]	71.57 ± 4.92	70.21 ± 5.20	1.562	0.120
**Score of questionnaires**				
NRS2002 [4]	1.05 ± 0.23	1.14 ± 0.40	−1.271	0.085
KPS [1]	86.40 ± 6.59	85.34 ± 7.06	1.005	0.316
CES-D [1]	7.87 ± 4.07	22.27 ± 5.25	−18.982	**<0.001**
SAS [1]	34.81 ± 4.67	46.45 ± 8.19	−10.331	**<0.001**

Notes: Data were shown as mean and standard deviation (SD) for continuous variables, and percentages (%) for categorical variables. [1] Independent Samples *t*-Tes*t*; [2] Chi-square test; [3] Chi-squared test with continuity correction; [4] Mann-Whitney test. DBC, depressed breast cancer patients; NBC, non-depressed breast cancer patients; BMI, body mass index; NRS2002, Nutritional Risk Screening 2002; KPS, Karnofsky performance status; CES-D, Center for Epidemiologic Studies Depression; SAS, Self-rating Anxiety Scale.

Table S3. Correlation between CES-D score and nutrient intakes, CHEI component scores (n = 205)

**Variables**	**r**	***p*-Value**	**Variables**	**r**	***p*-Value**
Energy [1]	–0.187	**0.010**	Potassium [1]	–0.222	**0.002**
Protein [1]	–0.200	**0.006**	Iron [2]	–0.229	**0.002**
Dietary fiber [1]	–0.205	**0.005**	Zinc [1]	–0.201	**0.006**
Vitamin A [2]	–0.239	**0.001**	Selenium [1]	–0.252	**0.001**
Vitamin B2 [2]	–0.240	**0.001**	Manganese [2]	–0.186	**0.011**
Niacin [2]	–0.179	**0.014**	Tryptophan [1]	–0.168	**0.027**
Calcium [1]	–0.145	**0.048**	Total CHEI score [1]	–0.201	**0.007**
Phosphorus [1]	–0.254	**<0.001**	Fruits [2]	–0.155	**0.039**

Notes: CES-D, Center for Epidemiologic Studies Depression; CHEI, Chinese Healthy Eating Index.[1] Pearson correlation analysis; [2] Spearman correlation analysis.

Table S4. Plasma amino acids of the depressed and non-depressed breast cancer patients

**Amino acid (μmol/L)**	**NBC (n = 46)**	**DBC (n = 17)**	***t***	***p*-Value**
Tryptophan	43.37 ± 6.07	40.10 ± 4.39	2.023	**0.047**
Tyrosine	77.50 ± 9.42	70.12 ± 15.35	1.140	0.275
Valine	224.68 ± 18.39	222.17 ± 29.62	0.200	0.844
Phenylalanine	59.96 ± 7.21	61.49 ± 18.25	–0.219	0.830
Isoleucine	69.30 ± 8.52	72.22 ± 7.42	–0.701	0.495
Leucine	124.04 ± 12.98	126.19 ± 22.66	–0.230	0.822
TRP/LNAAs	0.078 ± 0.010	0.076 ± 0.018	0.266	0.798

Notes: Data were shown as mean and standard deviation (SD) for continuous variable. DBC, depressed breast cancer patients; NBC, non-depressed breast cancer patients; TRP/LNAAs: tryptophan / large neutral amino acids.

Table S5. Differences of gut microbiota composition between the depressed and non-depressed breast cancer patients at the phylum level.

Species name	DBC		NBC		*p*-Value	Corrected *p*-Value	Lower ci	Upper ci	Effect size
	Mean (%)	SD (%)	Mean (%)	SD (%)					
p__Firmicutes	52.91	26.4	69.05	22.92	0.028	0.196	-30.32	-3.399	-16.14
p__Proteobacteria	31.68	29.13	12.6	21.35	0.01114	0.156	4.209	35.36	19.09
p__Actinobacteriota	7.283	7.953	9.285	11.86	0.6345	0.6874	-7.255	3.17	-2.002
p__Bacteroidetes	7.073	9.377	8.791	14.67	0.572	0.6874	-8.237	4.774	-1.718
p__Fusobacteriota	0.866	3.371	0.01561	0.04069	0.4105	0.6874	-0.01593	2.52	0.8504
p__Cyanobacteria	0.007777	0.02551	0.1195	0.6685	0.3024	0.6874	-0.3372	0.009154	-0.1117
p__Patescibacteria	0.08225	0.1315	0.04001	0.05147	0.364	0.6874	-0.01365	0.112	0.04223
p__Desulfobacterota	0.05969	0.1327	0.03566	0.06306	0.5383	0.6874	-0.0289	0.09839	0.02403
p__Verrucomicrobiota	0.01944	0.06822	0.04663	0.1565	0.4126	0.6874	-0.08971	0.02777	-0.02719
p__unclassified_k__norank_d__Bacteria	0.008944	0.01933	0.008891	0.01569	0.5426	0.6874	-0.00897	0.01137	5.27E-05
p__Synergistota	0.0003889	0.00165	0.0035	0.01066	0.1831	0.6874	-0.007189	-0.0001682	-0.003111
p__Campilobacterota	0.001361	0.005774	0.0003784	0.001376	0.7957	0.7957	-0.0007568	0.003894	0.0009827
p__Caldatribacteriota	0.0003889	0.00165	0.0002838	0.001726	0.6383	0.6874	-0.0008514	0.001167	0.0001051
p__Chloroflexi	0	0	0.0003784	0.002301	0.5101	0.6874	-0.001135	0	-0.0003784

Notes: Wilcoxon rank-sum test was used corrected for multiple testing using the Benjamini-Hochberg FDR method. DBC, depressed breast cancer patients (n = 18); NBC, non-depressed breast cancer patients (n = 37).

Table S6. Differences of gut microbiota composition between the depressed and non-depressed breast cancer patients at the genus level.

Species name	DBC		NBC		*p*-Value	Corrected *p* -Value	Lower ci	Upper ci	Effect size
	Mean (%)	SD (%)	Mean (%)	SD (%)					
g__*Escherichia-Shigella*	27.14	28.2	9.428	19.31	0.009772	0.2065	4.719	32.44	17.71
g__*Blautia*	9.127	8.672	16.75	13.9	0.02674	0.3131	-13.15	-1.582	-7.624
g__*Streptococcus*	5.857	8.626	5.102	9.548	0.6931	0.8695	-4.528	6.138	0.7551
g__*Eubacterium_hallii_group*	4.415	7.645	5.973	6.28	0.08012	0.441	-5.256	2.559	-1.558
g__*Bacteroides*	5.687	9.002	3.651	6.674	0.7265	0.8706	-2.139	6.769	2.036
g__*Bifidobacterium*	3.574	4.397	5.644	10.64	0.6731	0.8559	-6.365	1.371	-2.07
g__*Faecalibacterium*	4.029	7.084	4.957	6.375	0.1341	0.5454	-4.708	3.111	-0.9285
g__*Subdoligranulum*	3.747	6.597	2.861	3.653	0.3148	0.6833	-1.856	4.088	0.886
g__*Romboutsia*	4.18	6.009	2.023	2.697	0.9499	1	-0.6962	5.272	2.156
g__*Prevotella*	0.8341	1.719	3.849	10.67	0.9928	1	-6.933	-0.04011	-3.015
g__*Collinsella*	2.586	5.11	1.698	2.524	0.6131	0.8238	-1.077	3.809	0.8887
g__*unclassified_f__Lachnospiraceae*	1.915	4.043	1.685	1.619	0.2622	0.6429	-1.176	2.52	0.2306
g__*Haemophilus*	2.109	8.887	1.249	4.452	0.7217	0.8706	-2.279	5.742	0.8596
g__*Anaerostipes*	1.026	2.339	2.278	3.775	0.04449	0.3379	-2.914	0.4128	-1.252

g__Agathobacter	2.021	4.946	1.266	2.123	0.1455	0.5454	-1.231	3.374	0.7547
g__Ruminococcus	1.364	3.016	1.891	3.659	0.1034	0.501	-2.379	1.374	-0.5269
g__Megamonas	2.371	7.688	0.8753	4.484	0.9825	1	-1.823	5.644	1.496
g__Fusicatenibacter	1.116	2.136	1.699	3.511	0.1811	0.5786	-2.159	0.8525	-0.5831
g__Lactobacillus	0.6142	1.509	2.126	5.526	0.7186	0.8706	-3.593	0.1364	-1.512
g__Dorea	0.8526	0.8298	1.69	1.685	0.05725	0.3888	-1.507	-0.2176	-0.837
g__Enterococcus	1.853	4.341	0.59	2.287	0.8113	0.8905	-0.571	3.522	1.263
g__Lachnoclostridium	1.234	3.566	1.095	3.868	0.5184	0.7918	-1.89	2.283	0.1396
g__Klebsiella	1.375	3.572	0.79	2.948	0.3487	0.6999	-1.205	2.671	0.5852
g__Ruminococcus_torques_group	0.4276	0.6467	1.711	2.5	0.006054	0.189	-2.168	-0.5185	-1.283
g__Clostridium_sensu_stricto_1	0.5959	0.9136	0.9402	1.814	0.6203	0.8238	-1.14	0.3357	-0.3442
g__unclassified_f__Enterobacteriaceae	0.943	2.192	0.577	1.822	0.7003	0.8706	-0.7301	1.595	0.366
g__norank_f__Eubacterium_coprostanoligenes_group	0.1085	0.1879	1.332	2.727	0.003586	0.175	-2.183	-0.5007	-1.223
g__Monoglobus	0.3881	0.5445	0.9222	2.709	0.6214	0.8238	-1.619	0.1822	-0.5341
g__Lachnospiraceae_NK4A136_group	0.1064	0.1749	1.114	2.176	0.004625	0.175	-1.776	-0.3822	-1.007
g__Adlercreutzia	0.1935	0.3516	0.8825	1.503	0.003005	0.175	-1.243	-0.2103	-0.6891
g__Ruminococcus_gnavus_group	0.7105	1.369	0.3014	0.8533	0.1674	0.5534	-0.2289	1.184	0.4092
g__Coprococcus	0.2957	0.464	0.6013	0.9739	0.03911	0.3379	-0.6832	0.03363	-0.3056

g__Erysipelotrichaceae_UCG-003	0.125	0.2604	0.7705	1.624	0.01506	0.2341	-1.259	-0.2303	-0.6455
g__Fusobacterium	0.8637	3.371	0.01466	0.04071	0.2282	0.5883	-0.01605	2.444	0.849
g__Roseburia	0.4068	0.8952	0.3723	0.4644	0.123	0.5454	-0.3295	0.5375	0.03446
g__Alistipes	0.1563	0.3657	0.5304	1.435	0.08935	0.465	-0.9609	0.0251	-0.374
g__UCG-002	0.07641	0.1952	0.5955	1.129	0.00403	0.175	-0.8794	-0.1728	-0.5191
g__Phascolarctobacterium	0.1985	0.4243	0.454	1.903	0.3038	0.6775	-1.008	0.1962	-0.2556
g__Ruminococcus_gauvreauii_group	0.1674	0.2304	0.4335	0.8187	0.1749	0.5716	-0.5598	-0.02919	-0.2661
g__Intestinibacter	0.335	0.6981	0.2604	0.5451	0.6597	0.8426	-0.2455	0.4458	0.07458
g__Turicibacter	0.1814	0.3029	0.4055	1.86	0.5634	0.8202	-0.9024	0.2113	-0.224
g__Eggerthella	0.336	0.6117	0.1908	0.2391	0.7946	0.8803	-0.107	0.4361	0.1452
g__Dialister	0.4206	0.9778	0.09033	0.2204	0.7642	0.8775	-0.0558	0.8075	0.3302
g__norank_f__Coriobacteriales_Incertae_Sedis	0.1859	0.5472	0.3239	1.216	0.2827	0.6621	-0.6181	0.3028	-0.138
g__Christensenellaceae_R-7_group	0.0628	0.1048	0.4263	0.7082	0.02938	0.3232	-0.6089	-0.1438	-0.3635
g__Parabacteroides	0.2182	0.3662	0.2281	0.3405	0.2161	0.5883	-0.2094	0.2187	-0.01001
g__norank_f__Lachnospiraceae	0.1277	0.2394	0.3038	0.5503	0.06073	0.3888	-0.3905	0.01609	-0.1761
g__Butyricicoccus	0.1845	0.3041	0.2447	0.3239	0.1388	0.5454	-0.2205	0.1201	-0.06019
g__Holdemanella	0.008555	0.02516	0.3968	1.141	0.2801	0.6621	-0.8161	-0.07414	-0.3882
g__Erysipelatoclostridium	0.2535	0.6312	0.1174	0.3734	0.1459	0.5454	-0.1284	0.4644	0.1361

g__Veillonella	0.02994	0.04625	0.3279	1.69	0.6146	0.8238	-0.8747	0.0099	-0.2981
g__unclassified_f__Peptostreptococcaceae	0.1966	0.2731	0.16	0.1991	0.7732	0.8785	-0.1057	0.1759	0.03662
g__Hungatella	0.1589	0.3981	0.1738	0.5897	0.5428	0.8028	-0.2852	0.2484	-0.0149
g__Slackia	0.09099	0.3834	0.1968	0.8486	0.1034	0.501	-0.4803	0.1924	-0.1059
g__Lachnospiraceae_UCG-001	0.01517	0.03192	0.2278	0.7166	0.0299	0.3232	-0.4761	-0.03117	-0.2126
g__norank_f__Ruminococcaceae	0.03636	0.04694	0.1996	0.254	0.0002247	0.04863	-0.2539	-0.08807	-0.1632
g__Eubacterium_eligens_group	0.07602	0.2418	0.1587	0.4139	0.1414	0.5454	-0.2592	0.09478	-0.08271
g__unclassified_p__Firmicutes	0.126	0.2897	0.1086	0.2551	0.5835	0.8238	-0.1174	0.191	0.01742
g__norank_f__norank_o__Clostridia_UCG-014	0.03364	0.1045	0.1896	0.4861	0.1386	0.5454	-0.3158	-0.002462	-0.1559
g__Barnesiella	0.009916	0.03947	0.2108	0.9743	0.1079	0.5122	-0.5641	0.008839	-0.2009
g__Eubacterium_ventriosum_group	0.05327	0.1492	0.1621	0.3178	0.01175	0.2065	-0.2333	0.003694	-0.1088
g__Eisenbergiella	0.0453	0.1048	0.1587	0.5741	0.9567	1	-0.3452	0.03866	-0.1134
g__Parasutterella	0.09449	0.2676	0.09554	0.2263	0.1575	0.5454	-0.1331	0.1567	-0.001057
g__Lactococcus	0.09488	0.2683	0.09118	0.3923	0.775	0.8785	-0.178	0.1806	0.003705
g__Catenibacterium	0.1427	0.4295	0.03992	0.2049	0.1833	0.5786	-0.0602	0.3289	0.1028
g__Comamonas	0.0005833	0.001342	0.1748	0.879	0.6532	0.8381	-0.49	0.0006832	-0.1742
g__unclassified_o__Lactobacillales	0.07213	0.1874	0.09639	0.2678	0.8867	0.951	-0.1407	0.1054	-0.02425
g__Flavonifractor	0.09702	0.243	0.07094	0.1674	1	1	-0.07622	0.1643	0.02608

g__Paraprevotella	0.01089	0.03151	0.1472	0.553	0.1889	0.5786	-0.3391	-0.01416	-0.1363
g__Weissella	0.02392	0.06421	0.1314	0.7217	0.7341	0.8706	-0.3549	0.03468	-0.1075
g__Actinomyces	0.09508	0.1443	0.05798	0.07898	0.6215	0.8238	-0.02621	0.1206	0.03709
g__unclassified_f__Ruminococcaceae	0.05619	0.08342	0.09676	0.1219	0.03968	0.3379	-0.09431	0.01151	-0.04058
g__unclassified_c__Clostridia	0.04589	0.07677	0.102	0.1275	0.06442	0.4023	-0.1111	-0.00447	-0.05608
g__UBA1819	0.04278	0.1127	0.08579	0.1199	0.01583	0.2341	-0.1017	0.02598	-0.04302
g__norank_f__Oscillospiraceae	0.02586	0.08235	0.1015	0.1463	0.0003461	0.04863	-0.1433	-0.01645	-0.07564
g__norank_f__norank_o__Chloroplast	0.007777	0.02551	0.1195	0.6685	0.3024	0.6775	-0.3378	0.008462	-0.1117
g__Eubacterium_siraeum_group	0.0007777	0.002264	0.1261	0.3241	0.0327	0.3379	-0.2362	-0.03585	-0.1253
g__Family_XIII_AD3011_group	0.02664	0.03212	0.09989	0.1714	0.03538	0.3379	-0.1342	-0.02385	-0.07325
g__Marvinbryantia	0.03189	0.04391	0.077	0.08406	0.01739	0.2443	-0.08036	-0.01242	-0.04511
g__Eubacterium_brachy_group	0.01264	0.03429	0.08286	0.1519	0.01176	0.2065	-0.124	-0.02491	-0.07023
g__Eubacterium_xylanophilum_group	0.001167	0.004949	0.09374	0.3236	0.06088	0.3888	-0.2065	-0.01018	-0.09255
g__Alloprevotella	0.06339	0.2646	0.02998	0.09305	0.8404	0.9154	-0.05247	0.1694	0.03337
g__Senegalimassilia	0.04978	0.1285	0.04152	0.1682	0.7146	0.8706	-0.06961	0.08705	0.008252
g__NK4A214_group	0.0105	0.03013	0.07548	0.1254	0.0203	0.2717	-0.1125	-0.02349	-0.06499
g__Proteus	0.0009722	0.002892	0.08191	0.4959	0.7462	0.8706	-0.2446	0.001866	-0.08095
g__Lachnospiraceae_NC2004_group	0.03364	0.07174	0.04815	0.06788	0.08161	0.441	-0.04986	0.02718	-0.01451

g__unclassified_c__Gammaproteobacteria	0	0	0.08135	0.4948	0.5101	0.7918	-0.244	0	-0.08135
g__Tyzzerella	0.04005	0.1079	0.04105	0.1684	0.7059	0.8706	-0.0764	0.07473	-0.001
g__Rothia	0.06008	0.1257	0.01977	0.03502	0.1466	0.5454	-0.005676	0.1041	0.04031
g__Acinetobacter	0.0009722	0.001613	0.0787	0.474	0.5491	0.8078	-0.2341	0.0008934	-0.07773
g__Olsenella	0.008166	0.01176	0.07132	0.3148	0.2081	0.5883	-0.1721	0.005602	-0.06315
g__UCG-005	0.01283	0.03599	0.06583	0.1065	0.01009	0.2065	-0.0956	-0.019	-0.053
g__Odoribacter	0.01614	0.0449	0.05997	0.1934	0.1367	0.5454	-0.1115	0.01003	-0.04383
g__Granulicatella	0.04647	0.0617	0.02771	0.03807	0.2239	0.5883	-0.00794	0.05027	0.01875
g__Eubacterium_ruminantium_group	0.01011	0.03651	0.06328	0.1199	0.07683	0.4357	-0.1002	-0.01312	-0.05317
g__Bilophila	0.05386	0.114	0.01636	0.0286	0.6302	0.8287	-0.007599	0.09587	0.0375
g__Oscillibacter	0.02294	0.06796	0.04625	0.06723	0.07066	0.4317	-0.0568	0.02181	-0.02331
g__Lachnospira	0.01186	0.01825	0.05581	0.1406	0.2066	0.5883	-0.09831	-0.007794	-0.04395
g__Akkermansia	0.01944	0.06822	0.04654	0.1565	0.5185	0.7918	-0.08787	0.03001	-0.0271
g__Gordonibacter	0.01808	0.02882	0.04616	0.1295	0.9704	1	-0.07731	0.008776	-0.02808
g__Faecalitalea	0.008166	0.02297	0.05571	0.2413	0.2489	0.6301	-0.1373	0.005943	-0.04754
g__Clostridium_innocuum_group	0.03986	0.07207	0.02327	0.05101	0.9927	1	-0.01547	0.05915	0.01659
g__Sellimonas	0.02275	0.08162	0.03831	0.09326	0.4784	0.7918	-0.05967	0.03863	-0.01556
g__TM7x	0.03539	0.05719	0.02469	0.04277	0.481	0.7918	-0.01613	0.04279	0.0107

g__unclassified_f__Oscillospiraceae	0.002139	0.004006	0.0559	0.1881	0.008927	0.2065	-0.1204	-0.008913	-0.05376
g__CAG-352	0.02567	0.108	0.02828	0.1553	0.4857	0.7918	-0.07535	0.074	-0.002612
g__UCG-003	0.02197	0.04972	0.02762	0.05799	0.5345	0.8028	-0.03242	0.02284	-0.005649
g__Butyricimonas	0.02022	0.04947	0.02923	0.06318	0.07752	0.4357	-0.03689	0.02113	-0.009008
g__unclassified_f__Streptococcaceae	0.0245	0.04879	0.02431	0.1221	0.09238	0.472	-0.05213	0.04144	0.0001897
g__norank_f__norank_o__RF39	0.00175	0.007424	0.0455	0.1208	0.04308	0.3379	-0.08698	-0.01078	-0.04375
g__norank_f__Muribaculaceae	0.003305	0.01167	0.04124	0.1913	0.3237	0.6833	-0.1083	0.001051	-0.03794
g__Lachnospiraceae_UCG-004	0.003111	0.005354	0.0385	0.1429	0.256	0.6365	-0.08759	-0.004293	-0.03539
g__norank_f__Saccharimonadaceae	0.03247	0.06736	0.008513	0.009814	0.09428	0.4731	0.0005877	0.06232	0.02396
g__unclassified_o__Coriobacteriales	0.02703	0.1138	0.007	0.02267	0.2807	0.6621	-0.01229	0.07804	0.02003
g__Negativibacillus	0.01905	0.07997	0.01126	0.03373	0.2054	0.5883	-0.02023	0.0494	0.007798
g__Raoultibacter	0.0035	0.006684	0.02469	0.05721	0.03499	0.3379	-0.04415	-0.006406	-0.02119
g__Fructobacillus	0	0	0.02724	0.1657	0.5101	0.7918	-0.08173	0	-0.02724
g__CAG-56	0.007388	0.01113	0.01949	0.03084	0.2631	0.6429	-0.02422	-0.00206	-0.0121
g__Gemella	0.01769	0.03347	0.009081	0.0112	0.8616	0.9345	-0.004136	0.02486	0.008613
g__Fenollaria	0.01867	0.04225	0.00681	0.01942	0.2979	0.675	-0.005796	0.03396	0.01186
g__Desulfovibrio	0.005833	0.02306	0.01892	0.04702	0.1882	0.5786	-0.03091	0.00455	-0.01309
g__unclassified_f__Erysipelatoclostridiaceae	0	0	0.02431	0.1473	0.3331	0.6833	-0.07284	0	-0.02431

g__Mogibacterium	0.0007777	0.001919	0.02308	0.1292	0.04658	0.3445	-0.06526	-0.0001524	-0.0223
g__unclassified_c__Bacilli	0.0001944	0.0008249	0.02308	0.1345	0.5058	0.7918	-0.06773	9.98E-05	-0.02289
g__Lachnospiraceae_UCG-010	0.003889	0.009595	0.0193	0.07747	0.07373	0.4357	-0.04521	0.001403	-0.01541
g__Lachnospiraceae_ND3007_group	0.01342	0.02431	0.009743	0.01439	0.7482	0.8706	-0.006448	0.01663	0.003673
g__Rikenellaceae_RC9_gut_group	0.02314	0.09816	0	0	0.163	0.5454	0	0.06941	0.02314
g__Howardella	0.002917	0.009003	0.01911	0.1024	0.6397	0.8322	-0.05275	0.004483	-0.01619
g__Colidextribacter	0.001555	0.002994	0.02024	0.0685	0.6787	0.8591	-0.04496	-0.0004362	-0.01869
g__Sedimentibacter	0	0	0.02119	0.1289	0.5101	0.7918	-0.06357	0	-0.02119
g__norank_f__norank_o__Saccharimonadales	0.01303	0.03337	0.006716	0.01693	0.5738	0.8238	-0.006764	0.02507	0.006311
g__Eubacterium	0.001167	0.003395	0.01826	0.05088	0.2097	0.5883	-0.03507	-0.00412	-0.01709
g__norank_f__Eggerthellaceae	0.002139	0.005519	0.01646	0.03594	0.2948	0.6735	-0.02765	-0.003405	-0.01432
g__Solobacterium	0.014	0.02416	0.004162	0.008486	0.004981	0.175	0.001298	0.02372	0.009838
g__Porphyromonas	0.01322	0.04592	0.004919	0.01598	0.5547	0.8119	-0.006674	0.03382	0.008303
g__Lachnospiraceae_UCG-003	0.007	0.02481	0.01107	0.04127	0.6311	0.8287	-0.02251	0.01459	-0.004067
g__unclassified_k__norank_d__Bacteria	0.008944	0.01933	0.008891	0.01569	0.5426	0.8028	-0.009496	0.01134	5.26E-05
g__unclassified_f__Eggerthellaceae	0.004278	0.009719	0.01163	0.0319	0.7818	0.8799	-0.01899	0.002801	-0.007357
g__Atopobium	0.01128	0.01807	0.00454	0.01088	0.1601	0.5454	-0.001193	0.01683	0.006737
g__CHKCI002	0.0035	0.01485	0.01192	0.06571	0.7651	0.8775	-0.03368	0.008324	-0.008419

g__Peptoniphilus	0.007583	0.01515	0.007378	0.02135	0.648	0.8377	-0.009518	0.009522	0.0002048
g__Eubacterium_nodatum_group	0.009527	0.0171	0.005392	0.007676	0.918	0.9771	-0.002654	0.01344	0.004136
g__Prevotellaceae_NK3B31_group	0.009916	0.04207	0.00454	0.01718	0.5895	0.8238	-0.008796	0.02861	0.005376
g__Anaerococcus	0.007972	0.02343	0.005959	0.02983	0.3724	0.7421	-0.01322	0.01586	0.002012
g__Family_XIII_UCG-001	0.004083	0.01311	0.009081	0.02744	0.6871	0.8659	-0.01642	0.00503	-0.004997
g__Peptostreptococcus	0.008166	0.01067	0.004919	0.005801	0.5803	0.8238	-0.001503	0.009197	0.003248
g__Moryella	0.004278	0.01186	0.008513	0.02177	0.2673	0.6475	-0.01369	0.004451	-0.004235
g__Acidaminococcus	0	0	0.01258	0.07652	0.5101	0.7918	-0.03774	0	-0.01258
g__unclassified_f__Pasteurellaceae	0.005444	0.02138	0.006527	0.02417	0.398	0.7876	-0.01305	0.01108	-0.001083
g__Staphylococcus	0.004278	0.01563	0.007283	0.03905	0.8938	0.955	-0.01849	0.01033	-0.003006
g__Corynebacterium	0.006805	0.009021	0.004635	0.01281	0.1894	0.5786	-0.003868	0.00804	0.00217
g__norank_f__UCG-010	0	0	0.01126	0.03372	0.02499	0.3055	-0.02384	-0.002459	-0.01126
g__Holdemania	0.00175	0.004201	0.00927	0.01338	0.007685	0.2065	-0.01179	-0.003148	-0.00752
g__F0332	0.006611	0.0127	0.003594	0.005803	0.7828	0.8799	-0.002018	0.009727	0.003016
g__Candidatus_Stoquefichus	0.006611	0.02635	0.003594	0.01677	0.8647	0.9345	-0.008219	0.01829	0.003017
g__Scardovia	0.007194	0.02793	0.002365	0.004743	0.3377	0.6876	-0.003016	0.01929	0.004829
g__Coprobacillus	0.008166	0.02086	0.00123	0.003423	0.4714	0.7918	-0.0008092	0.01754	0.006937
g__Pediococcus	0	0	0.008513	0.04832	0.2271	0.5883	-0.02497	0	-0.008514

g__Candidatus_Soleaferrea	0.002917	0.004532	0.005486	0.007494	0.2858	0.6638	-0.005644	0.0005047	-0.00257
g__Delftia	0	0	0.008324	0.05004	0.3331	0.6833	-0.02488	0	-0.008324
g__Paraeggerthella	0	0	0.007756	0.03882	0.3331	0.6833	-0.02176	0	-0.007757
g__norank_f__norank_o__Clostridia_vadinBB60_group	0.007388	0.03135	0	0	0.163	0.5454	0	0.02217	0.007389
g__Aggregatibacter	0.003889	0.01025	0.003405	0.01784	0.5153	0.7918	-0.006958	0.007116	0.0004834
g__Enorma	0.0003889	0.001132	0.006905	0.02344	0.7216	0.8706	-0.01523	-8.94E-05	-0.006517
g__DTU089	0.0001944	0.0008249	0.007	0.01738	0.025	0.3055	-0.01248	-0.002076	-0.006806
g__Oribacterium	0.003694	0.01001	0.003121	0.007596	0.9635	1	-0.003668	0.006101	0.0005728
g__Pseudomonas	0	0	0.006716	0.04085	0.5101	0.7918	-0.02015	0	-0.006716
g__Christensenella	0.0009722	0.002632	0.005581	0.01108	0.08674	0.4599	-0.008508	-0.0009825	-0.004609
g__Megasphaera	0.001361	0.004006	0.005013	0.01855	0.8043	0.8863	-0.01047	0.001613	-0.003652
g__Finegoldia	0.002917	0.007424	0.003311	0.009931	0.347	0.6999	-0.004887	0.004241	-0.0003942
g__norank_f__Prevotellaceae	0.006027	0.02471	0	0	0.04353	0.3379	0	0.01789	0.006028
g__norank_f__Peptococcaceae	0.001167	0.004158	0.004824	0.01951	0.5984	0.8238	-0.01126	0.001471	-0.003657
g__Acetitomaculum	0.0001944	0.0008249	0.00577	0.0311	0.5173	0.7918	-0.01646	0.0001997	-0.005576
g__Bacillus	0	0	0.005959	0.03566	0.3331	0.6833	-0.01778	0	-0.00596
g__Morganella	0.005833	0.02475	9.46E-05	0.0005754	0.5993	0.8238	-0.0002838	0.0175	0.005739
g__norank_f__norank_o__Coriobacteriales	0.005055	0.01757	0.0008513	0.004623	0.4373	0.7918	-0.001608	0.0142	0.004204

g__*Sutterella*	0.001361	0.00497	0.004351	0.01247	0.2531	0.635	-0.007473	0.001324	-0.00299
g__*Peptococcus*	0.004083	0.01647	0.001419	0.004981	0.8404	0.9154	-0.002922	0.01091	0.002664
g__*Parvimonas*	0.003111	0.006225	0.002365	0.004522	0.7754	0.8785	-0.002071	0.004104	0.0007462
g__*Paludicola*	0.0003889	0.001132	0.004635	0.008654	0.04055	0.3379	-0.007	-0.001965	-0.004246
g__*Anaerotruncus*	0.0001944	0.0008249	0.00454	0.008202	0.002806	0.175	-0.007368	-0.001976	-0.004346
g__*Enterorhabdus*	0.0009722	0.004125	0.003405	0.02013	1	1	-0.009933	0.002728	-0.002433
g__*Anaerofustis*	0.0009722	0.003354	0.003311	0.01218	0.2091	0.5883	-0.007147	0.0009355	-0.002339
g__*Epulopiscium*	0.003889	0.01229	0.0001892	0.001151	0.194	0.5799	-0.0003784	0.01011	0.0037
g__*UCG-009*	0	0	0.003973	0.01204	0.05285	0.3714	-0.009082	-0.0009459	-0.003973
g__*Sporosarcina*	0	0	0.003689	0.02244	0.5101	0.7918	-0.01107	0	-0.003689
g__*Defluviitaleaceae_UCG-011*	0	0	0.003689	0.01027	0.05286	0.3714	-0.007189	-0.0008513	-0.003689
g__*Oxalobacter*	0	0	0.003689	0.01773	0.2271	0.5883	-0.01003	0	-0.003689
g__*Negativicoccus*	0.0009722	0.002632	0.002649	0.01611	0.07624	0.4357	-0.007556	0.001944	-0.001676
g__*Oscillospira*	0	0	0.003594	0.02186	0.5101	0.7918	-0.01079	0	-0.003595
g__*Frisingicoccus*	0.003111	0.0132	0.0003784	0.001804	0.9636	1	-0.0008514	0.009239	0.002733
g__*Allisonella*	0.0003889	0.001132	0.003027	0.01014	0.2917	0.672	-0.006327	-0.0001839	-0.002638
g__*Shuttleworthia*	0.003111	0.006225	0.0002838	0.0009685	0.01413	0.2336	0.0003994	0.006033	0.002827
g__*Ezakiella*	0.002139	0.006019	0.00123	0.003709	1	1	-0.001776	0.004104	0.0009092

g__Lachnospiraceae_FCS020_group	0.001361	0.003425	0.001986	0.005317	0.7287	0.8706	-0.002833	0.00185	-0.0006253
g__Catenibacillus	0.0009722	0.003354	0.002365	0.004953	0.1921	0.5799	-0.003568	0.0008093	-0.001393
g__Sneathia	0.002333	0.009899	0.0009459	0.004178	0.7957	0.8803	-0.001986	0.006811	0.001387
g__S5-A14a	0.002333	0.009899	0.0005675	0.002105	0.7957	0.8803	-0.00104	0.006622	0.001766
g__GCA-900066575	0.0001944	0.0008249	0.002554	0.006371	0.05881	0.3888	-0.00473	-0.0006516	-0.00236
g__Anaeroglobus	0.0001944	0.0008249	0.002459	0.0138	0.7348	0.8706	-0.007189	0.0003889	-0.002265
g__Cloacibacillus	0	0	0.002459	0.008646	0.1573	0.5454	-0.005676	-0.0001892	-0.002459
g__Fastidiosipila	0.002333	0.009899	0	0	0.163	0.5454	0	0.007	0.002333
g__norank_f__norank_o__MBA03	0.001361	0.005774	0.0009459	0.005194	1	1	-0.002554	0.004084	0.0004153
g__Stenotrophomonas	0	0	0.002176	0.01323	0.5101	0.7918	-0.006527	0	-0.002176
g__Gardnerella	0.0003889	0.00165	0.001703	0.007221	0.7051	0.8706	-0.004541	0.0007778	-0.001314
g__unclassified_o__Bacteroidales	0.0001944	0.0008249	0.001892	0.01039	1	1	-0.005297	0.0003942	-0.001697
g__norank_f__Christensenellaceae	0	0	0.002081	0.004323	0.01132	0.2065	-0.003595	-0.0009459	-0.002081
g__Burkholderia-Caballeronia-Paraburkholderia	0.00175	0.004688	0.0001892	0.0008023	0.1606	0.5454	-0.0001892	0.003904	0.001561
g__Pseudopropionibacterium	0.001361	0.002721	0.0005675	0.001937	0.2417	0.6174	-0.0005623	0.002239	0.0007935
g__norank_f__norank_o__Oscillospirales	0.0009722	0.003354	0.0009459	0.002281	0.5213	0.7918	-0.001324	0.002071	2.63E-05
g__Enhydrobacter	0	0	0.001892	0.01093	0.3331	0.6833	-0.005581	0	-0.001892
g__Propionibacterium	0.001167	0.002684	0.0006621	0.001993	0.4183	0.7918	-0.0007358	0.002049	0.0005045

g__Campylobacter	0.001361	0.005774	0.0003784	0.001376	0.7957	0.8803	-0.0007567	0.003989	0.0009827
g__Eubacterium_saphenum_group	0.0005833	0.001342	0.001135	0.002743	0.8782	0.9455	-0.001598	0.0005938	-0.0005518
g__Intestinimonas	0	0	0.001608	0.005509	0.1573	0.5454	-0.003595	-0.0001892	-0.001608
g__unclassified_f__Anaerovoracaceae	0	0	0.001608	0.009781	0.5101	0.7918	-0.004824	0	-0.001608
g__Harryflintia	0	0	0.001608	0.005809	0.1573	0.5454	-0.003689	-9.46E-05	-0.001608
g__Fournierella	0	0	0.001513	0.009206	0.5101	0.7918	-0.004541	0	-0.001514
g__Wolbachia	0.0007777	0.0033	0.0006621	0.002838	0.9818	1	-0.001419	0.001955	0.0001156
g__Pyramidobacter	0.0003889	0.00165	0.00104	0.003388	0.529	0.7992	-0.001892	0.0006096	-0.0006516
g__Brevundimonas	0.0001944	0.0008249	0.00123	0.006912	1	1	-0.0035	0.0003941	-0.001035
g__unclassified_f__Actinomycetaceae	0.0005833	0.001801	0.0007567	0.001869	0.6499	0.8377	-0.001135	0.0009827	-0.0001734
g__Anoxybacillus	0.0001944	0.0008249	0.00104	0.005208	0.7348	0.8706	-0.002838	0.0003889	-0.0008461
g__norank_f__Neisseriaceae	0.001167	0.004949	0	0	0.163	0.5454	0	0.0035	0.001167
g__Allorhizobium-Neorhizobium-Pararhizobium-Rhizobium	0	0	0.001135	0.005778	0.2271	0.5883	-0.003122	0	-0.001135
g__UCG-007	0	0	0.00104	0.005208	0.2271	0.5883	-0.002743	0	-0.001041
g__Hydrogenoanaerobacterium	0.0007777	0.0033	0.0001892	0.001151	0.5993	0.8238	-0.0003784	0.002333	0.0005886
g__Aerococcus	0	0	0.0009459	0.005194	0.3331	0.6833	-0.002743	0	-0.0009459
g__unclassified_o__Saccharimonadales	0.0007777	0.002562	9.46E-05	0.0005754	0.2019	0.5883	-0.0001892	0.002139	0.0006831
g__Murdochiella	0.0003889	0.00165	0.0004729	0.001874	0.7651	0.8775	-0.0009459	0.0008829	-8.41E-05

g__Serratia	0.0003889	0.001132	0.0004729	0.001467	1	1	-0.0007568	0.0006884	-8.41E-05
g__Leuconostoc	0.0005833	0.001801	0.0001892	0.0008023	0.4371	0.7918	-0.0002838	0.001366	0.0003941
g__Parascardovia	0.0005833	0.001342	0.0001892	0.0008023	0.1826	0.5786	-0.0001892	0.001072	0.0003941
g__Lachnoanaerobaculum	0.0001944	0.0008249	0.0005675	0.001308	0.2769	0.6621	-0.0009407	0.0002049	-0.0003731
g__Mobiluncus	0.0001944	0.0008249	0.0005675	0.001753	0.5172	0.7918	-0.001135	0.0002943	-0.0003731
g__norank_f__norank_o__Bacteroidales	0	0	0.0007567	0.004053	0.3331	0.6833	-0.002176	0	-0.0007568
g__Tsukamurella	0	0	0.0007567	0.004603	0.5101	0.7918	-0.00227	0	-0.0007568
g__norank_f__norank_o__norank_c__RBG-16-55-12	0.0005833	0.002475	9.46E-05	0.0005754	0.5993	0.8238	-0.0002838	0.00175	0.0004888
g__Candidatus_Caldatribacterium	0.0003889	0.00165	0.0002838	0.001726	0.6383	0.8322	-0.0008513	0.001167	0.0001051
g__Anaerofilum	0	0	0.0006621	0.001814	0.1094	0.5122	-0.001324	-9.46E-05	-0.0006621
g__unclassified_o__Oscillospirales	0	0	0.0006621	0.002157	0.1573	0.5454	-0.001419	0	-0.0006622
g__Candidatus_Saccharimonas	0.0005833	0.001801	0	0	0.04353	0.3379	0	0.001361	0.0005833
g__Cryptobacterium	0.0005833	0.001801	0	0	0.04353	0.3379	0	0.001556	0.0005833
g__Mycobacterium	0.0005833	0.002475	0	0	0.163	0.5454	0	0.00175	0.0005833
g__Acetanaerobacterium	0.0003889	0.001132	0.0001892	0.0008023	0.4607	0.7918	-0.0002838	0.0008776	0.0001997
g__unclassified_f__Christensenellaceae	0.0001944	0.0008249	0.0003784	0.001102	0.5403	0.8028	-0.0006621	0.0003889	-0.0001839
g__Clavibacter	0.0001944	0.0008249	0.0003784	0.001376	0.7348	0.8706	-0.0007567	0.0003942	-0.0001839
g__norank_f__Prolixibacteraceae	0.0003889	0.00165	9.46E-05	0.0005754	0.5993	0.8238	-0.0002838	0.001167	0.0002943

g__norank_f__Bacteroidales_UCG-001	0.0003889	0.00165	9.46E-05	0.0005754	0.5993	0.8238	-0.0001892	0.001167	0.0002943
g__Anaerostignum	0.0001944	0.0008249	0.0002838	0.0009685	0.7497	0.8706	-0.0005676	0.0003941	-8.94E-05
g__Dielma	0.0001944	0.0008249	0.0002838	0.0009685	0.7497	0.8706	-0.0005676	0.0003941	-8.94E-05
g__Marmoricola	0.0001944	0.0008249	0.0002838	0.0009685	0.7497	0.8706	-0.0005624	0.0003941	-8.94E-05
g__Phocea	0	0	0.0004729	0.001874	0.2271	0.5883	-0.001135	0	-0.000473
g__Coprobacter	0	0	0.0004729	0.001467	0.1572	0.5454	-0.000946	-9.46E-05	-0.000473
g__Macrococcus	0.0001944	0.0008249	0.0001892	0.0008023	1	1	-0.0003784	0.0004888	5.26E-06
g__Coriobacteriaceae_UCG-002	0	0	0.0003784	0.002301	0.5101	0.7918	-0.001135	0	-0.0003784
g__Brucella	0	0	0.0003784	0.001804	0.3331	0.6833	-0.001041	0	-0.0003784
g__DNF00809	0	0	0.0003784	0.001605	0.333	0.6833	-0.0009459	0	-0.0003784
g__norank_f__norank_o__Rhodospirillales	0	0	0.0003784	0.002301	0.5101	0.7918	-0.001135	0	-0.0003784
g__norank_f__Desulfovibrionaceae	0	0	0.0003784	0.002301	0.5101	0.7918	-0.001135	0	-0.0003784
g__norank_f__Clostridium_methylpentosum_group	0	0	0.0003784	0.002301	0.5101	0.7918	-0.001135	0	-0.0003784
g__Caldicoprobacter	0.0001944	0.0008249	9.46E-05	0.0005754	0.6186	0.8238	-0.0002838	0.0005834	9.99E-05
g__Lawsonella	0.0001944	0.0008249	9.46E-05	0.0005754	0.6186	0.8238	-0.0002838	0.0005833	9.98E-05
g__Merdibacter	0.0001944	0.0008249	9.46E-05	0.0005754	0.6186	0.8238	-0.0002838	0.0005833	9.98E-05
g__Eggerthia	0.0001944	0.0008249	9.46E-05	0.0005754	0.6186	0.8238	-0.0002838	0.0005834	9.99E-05
g__unclassified_f__Comamonadaceae	0	0	0.0002838	0.001726	0.5101	0.7918	-0.0008513	0	-0.0002838

g__norank_f__norank_o__RBG-13-54-9	0	0	0.0002838	0.001726	0.5101	0.7918	-0.0008513	0	-0.0002838
g__Pseudarthrobacter	0	0	0.0002838	0.0009685	0.2269	0.5883	-0.0006621	0	-0.0002838
g__Cellulosilyticum	0.0001944	0.0008249	0	0	0.163	0.5454	0	0.0005833	0.0001944
g__Lentimicrobium	0.0001944	0.0008249	0	0	0.163	0.5454	0	0.0005834	0.0001945
g__Parvibacter	0	0	0.0001892	0.001151	0.5101	0.7918	-0.0007568	0	-0.0001892
g__Bulleidia	0	0	0.0001892	0.001151	0.5101	0.7918	-0.0007567	0	-0.0001892
g__Empedobacter	0	0	0.0001892	0.001151	0.5101	0.7918	-0.0005676	0	-0.0001892
g__norank_f__Erysipelotrichaceae	0	0	9.46E-05	0.0005754	0.5101	0.7918	-0.0002838	0	-9.46E-05
g__norank_f__norank_o__norank_c__Anaerolineae	0	0	9.46E-05	0.0005754	0.5101	0.7918	-0.0002838	0	-9.46E-05
g__Johnsonella	0	0	9.46E-05	0.0005754	0.5101	0.7918	-0.0002838	0	-9.46E-05
g__norank_f__Bacteroidetes_vadinHA17	0	0	9.46E-05	0.0005754	0.5101	0.7918	-0.0003784	0	-9.46E-05
g__norank_f__UCG-011	0	0	9.46E-05	0.0005754	0.5101	0.7918	-0.0002838	0	-9.46E-05
g__unclassified_f__Rhizobiaceae	0	0	9.46E-05	0.0005754	0.5101	0.7918	-0.0002838	0	-9.46E-05
g__Victivallis	0	0	9.46E-05	0.0005754	0.5101	0.7918	-0.0002838	0	-9.46E-05
g__norank_f__Flavobacteriaceae	0	0	9.46E-05	0.0005754	0.5101	0.7918	-0.0002838	0	-9.46E-05

Notes: Wilcoxon rank-sum test was used corrected for multiple testing using the Benjamini-Hochberg FDR method. DBC, depressed breast cancer patients (n = 18); NBC, non-depressed breast cancer patients (n = 37).

Table S7. Linear regression analyses of the associations between diet and gut microbiota (n = 55).

Variables	Chao index				Shannon index				Simpson index			
	R^2	β	95%CI	*P*	R^2	β	95%CI	*P*	R^2	β	95%CI	*P*
Protein	0.181	0.229	–0.177, 1.323	0.131	0.175	0.150	–0.004, 0.012	0.323	0.091	–0.084	–0.002, 0.001	0.594
Dietary fiber	0.138	0.021	–3.785, 4.306	0.897	0.169	0.132	–0.025, 0.060	0.411	0.104	–0.158	–0.012, 0.004	0.346
Vitamin A	0.195	0.265	–0.008, 0.137	0.082	0.177	0.156	0.000, 0.001	0.305	0.101	–0.139	0.000, 0.000	0.381
Vitamin B2	0.166	0.188	–11.999, 49.729	0.225	0.159	-0.053	–0.388, 0.275	0.731	0.111	0.176	–0.029, 0.100	0.270
Niacin	0.157	0.158	–1.876, 5.694	0.315	0.165	0.102	–0.027, 0.053	0.513	0.089	–0.071	–0.010, 0.006	0.664
Calcium	0.212	0.305	0.001, 0.135	**0.048**	0.236	0.316	0.000, 0.001	**0.038**	0.135	–0.248	0.000, 0.000	0.121
Phosphorus	0.214	0.313	0.002, 0.128	**0.044**	0.191	0.211	0.000, 0.001	0.176	0.098	–0.128	0.000, 0.000	0.432
Potassium	0.163	0.188	–0.011, 0.041	0.249	0.204	0.255	0.000, 0.000	0.111	0.117	–0.207	0.000, 0.000	0.217
Iron	0.172	0.217	–1.080, 5.512	0.182	0.209	0.267	–0.005, 0.064	0.094	0.139	–0.271	–0.012, 0.001	0.104
Zinc	0.194	0.268	–0.704, 10.502	0.085	0.225	0.295	–0.001, 0.116	0.054	0.152	–0.291	–0.022, 0.001	0.069
Selenium	0.198	0.275	–0.082, 1.604	0.076	0.252	0.347	0.002, 0.019	**0.022**	0.134	–0.246	–0.003, 0.000	0.124

Manganese	0.139	0.048	–8.350, 11.186	0.832	0.166	0.116	–0.066, 0.139	0.477	0.099	–0.137	–0.028, 0.012	0.419
Total CHEI score	0.248	0.387	0.683, 5.839	**0.014**	0.258	0.369	0.006, 0.061	**0.018**	0.221	–0.426	–0.013, –0.002	**0.008**

Notes: The linear regression models were used after adjusting by age, BMI, family monthly income, education level, menopausal status, marital status, employment, residence and SAS score. CHEI, Chinese Healthy Eating Index.
